# Preparation and Photophysical Study of Rhodamine–Perylenebisimide Electron Donor–Acceptor Dyad/Triads Containing Flexible Linkers

**DOI:** 10.3390/molecules31111859

**Published:** 2026-05-28

**Authors:** Xin Guan, Haotian Bai, Jianzhang Zhao, Yan Wan

**Affiliations:** 1 State Key Laboratory of Fine Chemicals, Frontiers Science Center for Smart Materials Oriented Chemical Engineering, School of Chemical Engineering, Dalian University of Technology, 2 Ling Gong Road, Dalian 116024, China; guanxin@mail.dlut.edu.cn; 2 College of Chemistry, Beijing Normal University, Beijing 100875, China; haotian.bai@mail.bnu.edu.cn

**Keywords:** Förster resonance energy transfer, intersystem crossing, perylenebisimide, spin–orbit coupling, triplet state

## Abstract

We report the synthesis and characterization of the photophysical characterization of a series of rhodamine (Rho)–perylenebisimide (PBI) electron donor–acceptor dyad/triads containing flexible alkyl spacers (ethylene or hexylene chains). Steady-state absorption and emission, femtosecond and nanosecond transient absorption (fs-TA and ns-TA), cyclic voltammetry, triplet–triplet energy transfer (TTET) experiments and DFT/TD-DFT calculations were combined to elucidate the excited-state dynamics. fs-TA spectral study indicates fast decay of the S_1_ state and formation of the ^3^PBI state (0.32–663 ps), which is supported by the ns-TA spectra. The localized PBI triplet (^3^PBI*) exhibits unusually long lifetimes (up to 272 μs) as determined by the TTET experiment. No long-lived charge-separated (CS) state was observed. While a Förster resonance energy transfer (FRET) probably occurs between PBI and the open-ring rhodamine, a photo-induced electron transfer is proposed to be responsible for the quenching of the fluorescence of the PBI moiety.

## 1. Introduction

Electron donor–acceptor compounds are of particular interest due to their potential applications in photocatalysis, photovoltaics, and organic light emitting diodes (OLEDs) [1,2,3,4,5]. These organic compounds are also significant in fundamental photochemistry studies, for instance, in the investigation of electron transfer (ET), charge separation (CS), charge recombination (CR) and intersystem crossing (ISC). For these organic compounds, CR-induced ISC is an attractive method for the design of heavy-atom-free triplet photosensitizers, i.e., to populate the localized triplet state (^3^LE) of a chromophore using a CS state as the precursor of the ^3^LE state [6,7].

CR-induced ISC has been known for decades in conventional electron donor–acceptor compounds. Usually, long and rigid spacers are used to separate the electron donor and electron acceptor units to prolong the CS state lifetime, and to mimic the photosynthesis system, but this makes the preparation of the compounds challenging. In such electron donor–acceptor dyads, one of the mechanisms for triplet state formation is radical pair ISC (RP-ISC) [8,9,10,11]. This process is primarily driven by hyperfine coupling (HFC), spin–orbit coupling (SOC), *g*–factor difference (Δ*g*), or exchange interaction (*J*). Although triplet state properties have been studied, the modulation of triplet quantum yields via molecular structure design, especially without invoking the heavy-atom effects, remains challenging [12,13,14,15,16]. Recently, the spin–orbit charge transfer ISC (SOCT-ISC) was developed; this CR-induced ISC is different from the traditional electron donor–acceptor dyads showing the RP–ISC mechanism. In the D-A compounds showing SOCT-ISC, the π-conjugated planes of the electron donor and electron acceptor moieties typically take a nearly orthogonal geometry [14,17], and direct linkages (e.g., a single bond) of the donor and acceptor moiety are used. Upon photoexcitation, strong electronic coupling facilitates the formation of an ultrafast CS state. Since the π-conjugated planes of the electron donor and electron acceptor are oriented orthogonally, the D-A molecules with mutually perpendicular π–orbitals may undergo a PET process upon light absorption. During the subsequent CR evolution, this specific perpendicular conformation induces a change in the orbital angular momentum of the electron. To conserve the total angular momentum of the system, the electron must undergo a corresponding spin flip. This facilitates ISC mediated by the charge recombination process. Furthermore, solvent polarity may modulate the relative energy levels, i.e., non-polar media maintain the CS state above T_1_ to favor triplet generation, whereas highly polar solvents stabilize the CS state, leading to its direct CR to the ground state [18,19,20,21].

This SOCT-ISC mechanism is characterized by fast kinetics and high triplet state yields. Recently, various electron donor–acceptor compounds showing the SOCT-ISC mechanism based on different visible light–absorbing chromophores have been developed, for instance, those based on boron–dipyrromethene (BODIPY) [22,23], perylene [24], perylenebisimide (PBI) [8,25,26], etc. In these dyads, the electron donor and electron acceptor are connected with a short, rigid linker, normally a single C–N or C–C bond, but the geometry is not fully fixed, so a certain extent of geometrical fluctuation is possible. We recently proposed using the sterically congested, closed-form (lactam form) of rhodamine as an electron donor, and effective SOCT–ISC was observed, which led to the efficient formation of the ^3^LE state (localized excited state) or the long-lived triplet CS (^3^CS) state [15]. However, it is clear that the molecular structure diversity of these electron donor–acceptor dyads needs to be extended to study the CS, CR and ISC processes, as well as the relationship between the molecular structure and the ISC efficiency.

**Scheme 1:**
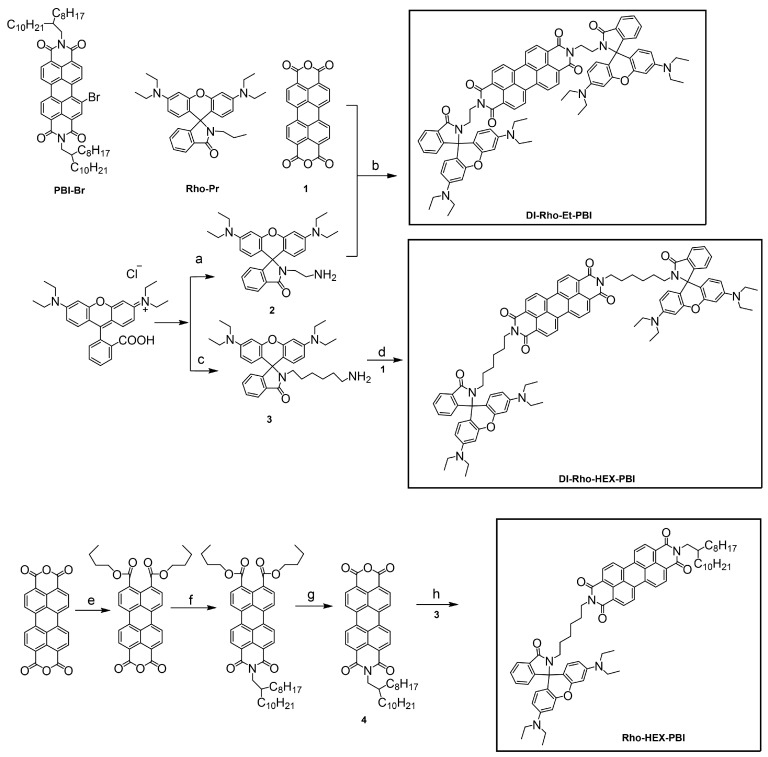
Key: (a) Ethylenediamine, ethanol, 90 °C, 12 h, yield: 62%; (b) Et_3_N, tripropylamine, DMF, 160 °C, 12 h, yield: 20%; (c) 2,6-hexamethylenediamine, 4-nitroaniline, 1,2-dichloroethane, phosphorus oxychloride, 120 °C, 12 h, yield: 44%; (d,h) are similar to (b); (d) yield: 22%; (h) yield: 10%; (e) DBU, *n*-Butanol, DMF, 60 °C, 0.5 h, yield: 37%; (f) 2-octyldodecylamine, imidazole, 160 °C, 5 h, yield: 33%; (g) glacial acetic acid, H_2_SO_4_, 100 °C, 2 h, yield: 36%.

Previously, our group investigated Rho-acceptor dyad molecular systems, in which the electron acceptor unit was rigidly and covalently linked to the electron donor [15]. As a comparative and extended study, this paper presents the design and synthesis of electron donor-acceptor dyad/triad where the electron donor and electron acceptor units are connected via flexible alkyl chains. This work aims to systematically investigate the influence of the linking method and molecular conformational flexibility on photophysical processes and the mechanisms of energy and electron transfer. Herein, we use the rhodamine unit as the electron donor and PBI as the electron acceptor, which is also the visible light-absorbing chromophore. One dyad and two triads were prepared (Scheme 1), ethylenediamine or hexamethylenediamine flexible linkers used to connect the electron donor and electron acceptor. The photophysical properties of the dyad and the triads, including the ISC process, were studied using steady-state and femtosecond/nanosecond transient absorption spectroscopic methods.

## 2. Results and Discussion

### 2.1. Molecular Structure Design Rationales

Rhodamine is a xanthene-based fluorescent dye that plays pivotal roles in bioimaging, fluorescence sensing, and photodynamic therapy studies [27,28,29]. Previous investigations on rhodamine derivatives have typically focused on their use as compact electron donors in electron donor–acceptor systems to investigate photophysical properties.

On the other hand, the formation of the localized ^3^LE state in electron donor–acceptor dyads showing SOCT-ISC usually relies on a rigid structure with orthogonal geometry between the electron donor and electron acceptor; studies of the SOCT–ISC effect in the electron donor–acceptor dyad containing flexible linkers are rare. Therefore, herein, we designed a series of electron donor–acceptor dyads and triads containing rhodamine/PBI units and different flexible linkers (Scheme 1). The synthesis of the compounds is based on the routine derivatization chemistry of the chromophores. The molecular structures of the compounds were confirmed by ^1^H NMR, ^13^C NMR, and high-resolution mass spectrometry (HRMS) (Figures S1–S13). In addition, we studied the changes in the ^1^H NMR spectra before and after adding deuterated trifluoroacetic acid (TFA-*d*); the color changed from light red to a prominent yellow fluorescence (Figures S14 and S15).

### 2.2. UV–Vis Absorption and Fluorescence Spectra

**Figure 1:**
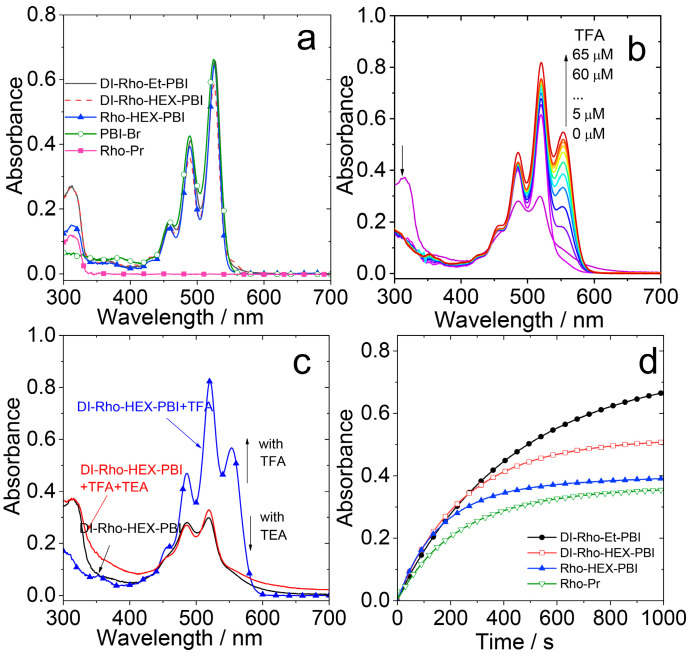
(**a**) UV–vis absorption spectra of the compounds in toluene (TOL), and the UV–vis absorption spectra of **DI-Rho-HEX-PBI** (**b**) with increasing amount of trifluoroacetic acid (TFA) added; (**c**) TFA (65 μL) was added first, followed by addition of organic base triethylamine (TEA, 300 μL); (**d**) kinetics of the lactam ⟶ opened amide transformation of Rho moiety for the dyad and the triads, monitored at 557 nm upon addition of TFA in MeOH. *c* = 1.0 × 10 ^−5^ M. 25 °C.

The UV–vis absorption spectra of the compounds are presented in Figure 1. **PBI-Br** was used as a reference compound due to its good solubility and ISC capability (without Br substitution at the bay position, no ISC capability exists for the PBI chromophore). **PBI-Br** shows the characteristic strong absorption band centered at 520 nm (Figure 1a). On the other hand, another reference compound, **Rho-Pr**, shows an absorption band centered at 310 nm. The absorption spectra of the dyads and triads are virtually the sum of the spectra of the individual components, demonstrating the lack of interactions between the electron donor and electron acceptor at ground state.

As illustrated in Figure 1a, all target compounds (**Rho-HEX-PBI**, **DI-Rho-HEX-PBI**, and **DI-Rho-Et-PBI**) exhibit intense absorption bands in the 400–550 nm range, primarily attributed to the characteristic π–π* transitions of the PBI moiety. No significant absorption was observed in the visible region beyond 550 nm, indicating that the rhodamine (Rho) units predominantly exist in the colorless, closed-ring spirolactam form.

The reversible transformation of the Rho moiety between the closed lactam and the open-ring xanthene form was studied via acid/base-induced spectroscopic titrations (Figure 1b,c). To quantify this process, the ring-opening kinetics were continuously monitored at 557 nm upon the addition of TFA in MeOH (Figure 1d). The model donor **Rho-Pr** exhibited the slowest transformation kinetics with an apparent rate constant of *k* = 3.5 × 10^−3^ s^−1^. Upon connection of the rhodamine unit to the PBI electron acceptor, the rate constants increased slightly: **Rho-HEX-PBI** shows *k* = 3.9 × 10^−3^ s^−1^, while the di-rhodamine systems **DI-Rho-HEX-PBI** and **DI-Rho-Et-PBI** displayed slightly faster kinetics *k* of 5.1 × 10^−3^ s^−1^ and *k* = 6.7 × 10^−3^ s^−1^, respectively. This acceleration suggests that the presence of the multiple Rho units introduces steric hindrance that may affect the transformation kinetics. Compounds **DI-Rho-Et-PBI** and **Rho-HEX-PBI** were found to exhibit similar properties (Figure S16).

Interestingly, the ring-opening kinetics observed in our PBI-based systems are approximately two to three orders of magnitude slower than those reported for structural analogues naphthalenediimide (NDI) –based dyads (*k* = 10^−1^~10^0^ s^−1^) under similar conditions. This difference may be rationalized by the electronic nature of the electron acceptor; PBI is a significantly stronger electron acceptor than NDI [30], as evidenced by its more positive reduction potential. Furthermore, although linker length and conformational flexibility exert a limited impact on the macroscopic reaction rate, they may play a potential regulatory role in modulating the photophysical property of the dyads and the triads.

**Figure 2:**
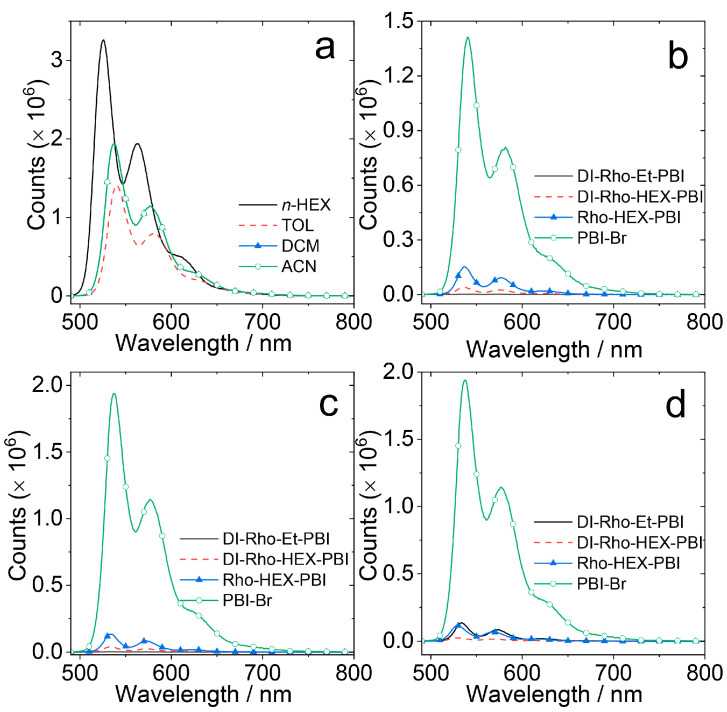
Fluorescence emission spectra of (**a**) **PBI-Br** in different solvents. Fluorescence emission spectra of the compounds in (**b**) TOL, (**c**) DCM and (**d**) ACN. Optically matched solutions were used (all the solutions show the same absorbance at the excitation wavelength, *λ*_ex_ = 515 nm, *A*_515 nm_ = 0.1) 25 °C.

Regarding the photophysical behavior, the fluorescence spectra of the compounds were compared with that of the reference **PBI-Br** (Figure 2). The reference **PBI-Br** exhibited weakly solvent polarity-dependent fluorescence intensity, with its fluorescence intensity decreasing as the solvent polarity increased (Figure 2a). In contrast, the target compounds showed drastic fluorescence quenching across solvents of varying polarities, including toluene (TOL), dichloromethane (DCM), and acetonitrile (ACN) (Figure 2b,d). These results propose a photo-induced electron transfer (PET) process in this compound upon photoexcitation, which is responsible for the fluorescence quenching of the dyad and triads in polar solvents.

**Figure 3:**
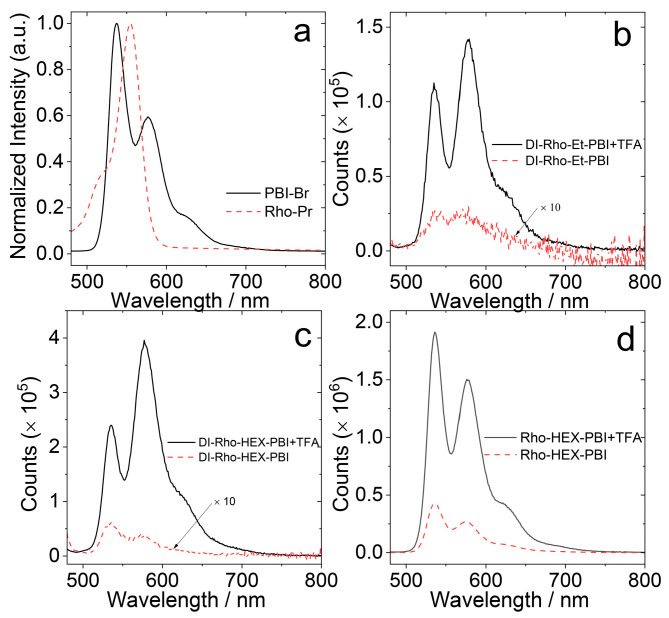
(**a**) Normalized spectral overlap between the emission of **PBI-Br** and the absorption of **Rho-Pr**. Fluorescence emission spectra of (**b**) **DI-Rho-Et-PBI,** (**c**) **DI-Rho-HEX-PBI** and (**d**) **Rho-HEX-PBI** in MeOH with increasing amount of trifluoroacetic acid (TFA, 65 μL) added. Optically matched solutions were used, *λ*_ex_ = 480 nm, *A*_480 nm_ = 0.5, 25 °C.

Although PBI units are typically used as electron acceptors in previous studies, they function as energy donors within the Förster resonance energy transfer (FRET) process discussed herein. Conversely, the Rho unit in the opened structure serves as the energy acceptor. The spectral overlap between the absorption spectrum of the open-ring **Rho-Pr** and the fluorescence emission spectrum of **PBI-Br** was investigated in ACN. A significant overlap was observed (Figure 3a), providing condition for the FRET. Upon photoexcitation, the PBI unit transfers its excited-state energy to the Rho moiety via long-range dipole–dipole interactions [31,32]. When the Rho moiety is in its closed form, the FRET is inhibited, and the quenching of the fluorescence is mainly due to PET. As the Rho ring opens in the presence of acid, a significant spectral overlap emerges between the energy acceptor’s absorption and the energy donor’s emission, thereby triggering an energy transfer process. Consequently, the energy donor fluorescence is quenched, accompanied by the sensitized emission of the energy acceptor [33].

Trifluoroacetic acid (TFA) was introduced to the solution to trigger the ring opening of the Rho moiety [27,28]. Upon adding 65 µL of TFA to the solutions of **DI-Rho-Et-PBI**, **DI-Rho-HEX-PBI** and **Rho-HEX-PBI** (Figure 3a,b), a significant attenuation of the characteristic emission peak of PBI (530 nm) was observed, concurrently with a corresponding enhancement of the sensitized emission peak of Rho (600 nm). This spectral evolution, characterized by the simultaneous enhancement of energy acceptor emission and depletion of energy donor emission, corroborates our hypothesis regarding the excited-state deactivation pathways.

Experimental results demonstrated that, compared to the fluorescence decay observed for the **PBI-Br** monomer [34], the energy donor lifetime in the D-A dyad was shortened. This short excited-state lifetime provides evidence for non-radiative decay of the S_1_ state, such as energy transfer or PET [35,36].

To investigate the excited-state deactivation pathways, we analyzed the fluorescence quantum yields (*Φ*_F_) and the singlet oxygen quantum yields (*Φ*_Δ_).. Significant fluorescence quenching was observed for all target compounds, with *Φ*_F_ values (0.2%~5.9%) drastically lower than the reference **PBI-Br** (77.1%). We found the singlet oxygen quantum yields are generally low (Table 1). The production of the trace triplet state is probably due to the CR. However, the mutual orientation of the PBI and the Rho units in the dyad and triads is not fixed; therefore, the CR-induced ISC is non-efficient (for instance, the situation is not optimized for the SOCT-ISC mechanism). While trace singlet oxygen generation was detectable in non-polar solvents (*Φ*_Δ_ = 1.1%~2.8%), *Φ*_Δ_ values decreased to zero in moderately and highly polar solvents such as DCM and ACN (Table 1 and Table S1). This decrease in *Φ*_F_ and *Φ*_Δ_ reveals a fast CR to the ground state to populate the ^3^PBI state.

### 2.3. Electrochemistry Study

**Figure 4:**
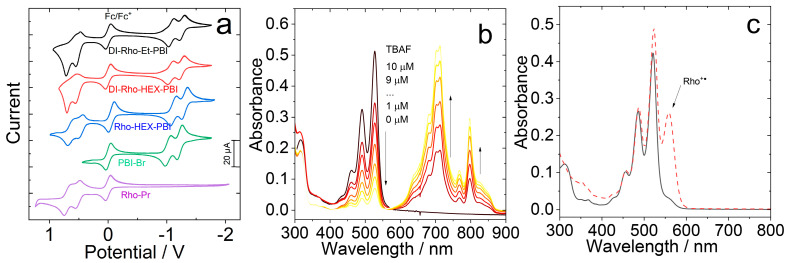
(**a**) Cyclic voltammogram of the compounds in DCM containing 0.10 M Bu_4_NPF_6_ as supporting electrolyte and with Ag/AgNO_3_ reference electrode. Working electrode: glassy carbon electrode; counter electrode: platinum wire. Scan rate: 100 mV/s. *c* = 1.0 × 10^−3^ M. Ferrocene (Fc) was used as internal reference, 20 °C. *c* = 1.0 × 10^−3^ M. (**b**) **DI-Rho-Et-PBI** chemically reduced with tetrabutylammonium fluoride (TBAF) used to generate PBI^−●^ in de-aerated DMF. UV–vis absorption spectra of **DI-Rho-Et-PBI** (**c**) chemically oxidized with nitrosonium hexafluorophosphate (NOPF_6_) in de-aerated ACN. In this case, the opened form of the dyad is produced. *c* = 1.0 × 10^−1^ M. 25 °C.

The electrochemical and spectroelectrochemical properties of compounds were studied va cyclic voltammograms and the radical cation and anions, with chemical oxidation/reduction (Figure 4). **DI-Rho-Et-PBI**, **DI-Rho-HEX-PBI**, and **Rho-HEX-PBI** exhibit two reversible reduction waves, with half–wave potentials of −1.27 and −1.08 V, −1.26 and −1.06 V, and −1.31 and −1.11 V (vs. Fc/Fc^+^), respectively. The reduction waves of **Rho-HEX-PBI** are more cathodically shifted than those of **DI-Rho-HEX-PBI**.

To facilitate the assignment of absorption of the radical anion and cations in the transient absorption spectra (see later section), the radical anions and cations of the dyad and triads were generated via chemical reduction/oxidation titrations. For **DI-Rho-Et-PBI**, reduction with tetrabutylammonium fluoride (TBAF) resulted in the neutral absorption bands at 320 and 520 nm disappearing, accompanied by the emergence of new near-infrared peaks at 710 and 800 nm (Figure 4b). These new absorption bands are characteristic of the PBI^−●^. Similar spectral transformations were observed for **DI-Rho-HEX-PBI** and **Rho-HEX-PBI** upon both TBAF reduction and the application of cathodic potential (−1.2 V. Figure S18). Conversely, oxidation of **DI-Rho-Et-PBI** using nitrosonium hexafluorophosphate (NO[PF_6_]) triggered the appearance of new absorption bands at 520 and 560 nm (Figure 4c). These signals agree well with the Rho^+●^ absorption previously identified for **Rho-NDI** through spectroelectrochemistry and TD-DFT calculation [37,38]. These steady-state spectral signatures provide essential benchmarks for identifying the possible CS state species in subsequent transient absorption studies.

The results indicate that the reference compound **Rho–Pr** exhibits a reversible oxidation wave at +0.51 V, while the PBI electron acceptor units show a significantly more positive reduction potential (*E*_red_) compared to NDI-based systems, confirming the strong electron-accepting capability of PBI. In the Rho-PBI dyad, the simultaneous presence of oxidation potentials attributed to the Rho moiety and reduction potentials attributed to the PBI moiety confirms that within this electron donor–acceptor (D–A) architecture, Rho acts as the electron donor while PBI functions as the electron acceptor. It was observed that subtle structural modifications significantly influence the electrochemical behavior. Compared to the dyad, the triad (**DI-Rho-Et-PBI**, **DI-Rho-HEX-PBI**) exhibits a slight shift in reduction potential due to the addition of an extra Rho unit. Furthermore, while the introduction of flexible linkers (e.g., transitioning from ethyl to hexyl) has a limited direct impact on the redox center potentials, it markedly modulates the Coulombic interaction term during charge separation by varying the effective distance between the electron donor and electron acceptor.

Energies of the charge-separated states (*E*_CS_) can be calculated with Equation (1). The Gibbs free energy changes for the intramolecular electron transfer process (Δ*Gs*) can be calculated with the Rehm–Weller equation (Equations (2) and (3)). The data are shown in Table 2.

(1) $$E_{CS}=e\left[E_{OX}-E_{RED}\right]+\Delta G_{S}$$

(2) $$\Delta G_{S}=-\frac{e^{2}}{4\pi \varepsilon_{S}\varepsilon_{0}R_{CC}}-\frac{e^{2}}{8\pi \varepsilon_{0}}\left(\frac{1}{R_{D}}+\frac{1}{R_{A}}\right)\left(\frac{1}{\varepsilon_{REF}}-\frac{1}{\varepsilon_{S}}\right)$$

(3) $$\Delta G_{CS}^{0}=e\left[E_{OX}-E_{RED}\right]-E_{00}+\Delta G_{S}$$

The Gibbs free energy changes for PET ($\Delta G_{CS}^{0}$), calculated using the Rehm–Weller equation (Equation (4)), demonstrate that the PET is thermodynamically allowed for all dyads and triads [39,40,41]. However, for **DI-Rho-Et-PBI**, in both non-polar and polar solvents, the CS state energy is higher than the triplet energy of the ^3^PBI* state (ca. 1.20 eV), which is in agreement with the observation of the singlet oxygen generation (*Φ*_Δ_ = 1.1%~2.8%). However, this result also suggests that the CS state may not be observed, due to the possible fast CR to give the ^3^PBI state and the S_0_ state. In polar solvents, the singlet oxygen quantum yield (*Φ*_Δ_) is close to zero, indicating that fast CR directly to the S_0_ state may completely inhibit the CR-induced ISC.

### 2.4. Nanosecond Transient Absorption (ns-TA) Spectra

**Figure 5:**
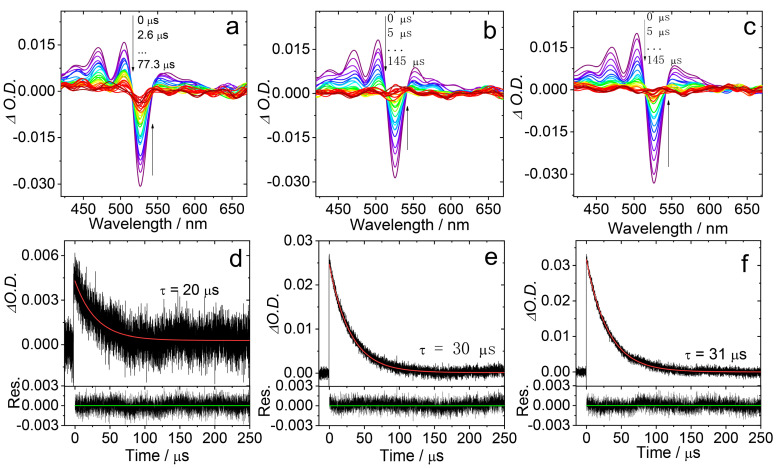
Nanosecond transient absorption spectra of (**a**) **DI-Rho-Et-PBI** in de-aerated TOL/CH_3_I (5:1, *v*/*v*) mixed solvent, *c* = 1.0 × 10^−5^ M, (**b**) **DI-Rho-HEX-PBI** in de-aerated TOL/CH_3_I (5:1, *v*/*v*) mixed solvent, *c* = 1.0 × 10^−5^ M (**c**) **Rho-HEX-PBI** in de-aerated TOL/CH_3_I (5:1, *v*/*v*) mixed solvent, *c* = 1.0 × 10^−5^ M. The corresponding decay traces of (**d**) **DI-Rho-Et-PBI** at 505 nm, (**e**) **DI-Rho-HEX-PBI** at 505 nm, (**f**) **Rho-HEX-PBI** at 505 nm. *λ*_ex_ = 520 nm, 20 °C. Laser energy is 12 mJ per pulse.

Our investigation revealed that all target compounds exhibit singlet oxygen photosensitizing ability in non-polar solvents; thus, the ns-TA spectra measurements were performed in non-polar media. However, for Rho-PBI derivatives, significant ns-TA signals were only observable upon the addition of methyl iodide to induce the heavy-atom effect [42,43]. When methyl iodide is used as the solvent, the external heavy-atom effect of the iodine atoms enhances the SOC, which leads to a acceleration in the otherwise negligible ISC. To further evaluate the triplet state properties of the current compounds, ns-TA spectra were recorded in a toluene/methyl iodide (5:1, *v*/*v*) mixture (Figure 5). For **DI-Rho-Et-PBI** (Figure 5a), a ground state bleaching (GSB) band centered at 520 nm was observed, consistent with the steady-state absorption spectra (Figure 1). Simultaneously, two broad ESA bands were observed in the ranges of 460–520 nm and 550–620 nm, which are characteristic of the ^3^PBI* state absorption. These results indicate that the lowest ^3^LE state of **DI-Rho-Et-PBI** is localized on the PBI moiety. The triplet lifetime was measured as 126 μs [34]. As illustrated in Figure 5d–f, dyad/triads systems—**DI-Rho-Et-PBI**, **DI-Rho-HEX-PBI**, and **Rho-HEX-PBI**—exhibit shorter triplet lifetimes compared to un-substituted PBI (126 μs).

**Figure 6:**
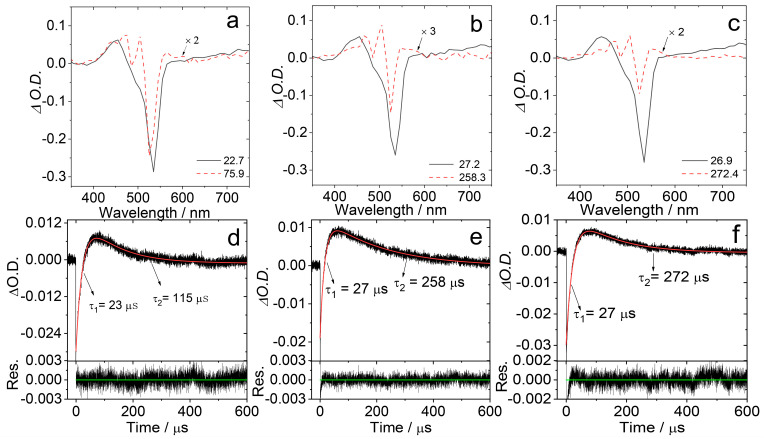
Intermolecular triplet–triplet energy transfer (TTET) with **2I-BDP** (*c* = 2.5 × 10^−6^ M) as the triplet photosensitizer and different compounds as the triplet energy acceptor in TOL, *λ*_ex_ = 520 nm. (**a**) **DI-Rho-Et-PBI**, (**b**) **DI-Rho-HEX-PBI** and (**c**) **Rho-HEX-PBI** species-associated difference spectra (SADS) of nanosecond transient absorption spectra of TTET obtained by global fitting (sequential model), (**d**) **DI-Rho-Et-PBI** (**e**) **DI-Rho-HEX-PBI** and (**f**) **Rho-HEX-PBI** decay traces monitored at 520 nm, *c* = 1.0 × 10^−5^ M 25 °C.

To study the triplet state properties of the dyad/triads in polar solvents, **2I-BDP** was employed as a triplet photosensitizer (PS). The triplet energy of **2I-BDP** (*E*_T1_ = 1.60 eV) is similar to the calculated ^3^LE state energy of PBI (1.60 eV) and the CT state energy. Upon excitation of the mixed solution in de-aerated TOL, the GSB and ESA bands of **2I-BDP** were observed instantaneously; however, the lifetime of the **2I-BDP** was shortened from 186 μs (pristine) to 27 μs in the mixture, confirming an efficient intermolecular triplet–triplet energy transfer (TTET) process. Similar experiments give apparent triplet lifetimes of 115 μs, 258 μs, and 272 μs for **DI-Rho-Et-PBI**, **DI-Rho-HEX-PBI**, and **Rho-HEX-PBI**, respectively (Figure 6d, Figure 6e and Figure 6f). These results indicate that the lack of a triplet signal for the dyad/triads in polar solvents is not due to the shorter triplet state lifetime; instead, it is due to the non-efficient ISC. This finding provides evidence for the advantages of utilizing the heavy-atom-free ISC mechanism to access long-lived localized ^3^LE. Long-lived triplet states are critical for applications in photodynamic therapy (PDT), photocatalysis, and triplet–triplet annihilation (TTA) upconversion.

### 2.5. Femtosecond Transient Absorption (fs-TA) Spectra

**Figure 7:**
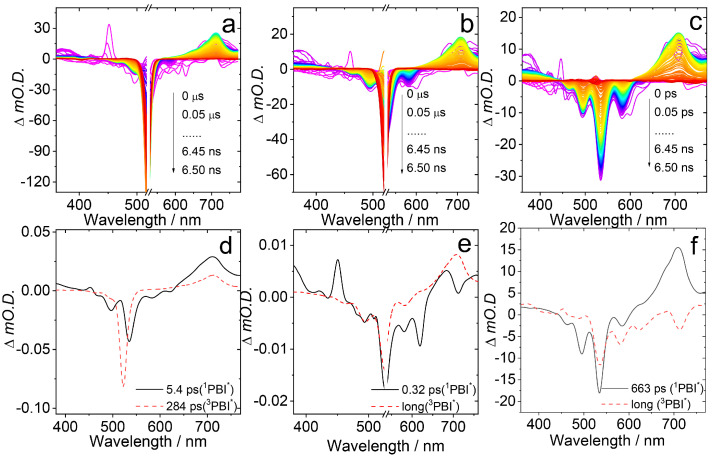
Femtosecond transient absorption spectra of (**a**) **DI-Rho-Et-PBI**, (**b**) **DI-Rho-HEX-PBI** and (**c**) **Rho-HEX-PBI** in TOL. Evolution associated difference spectra (EADS) obtained from global analysis of time resolved data in TOL for (**d**) **DI-Rho-Et-PBI**, (**e**) **DI-Rho-HEX-PBI** and (**f**) **Rho-HEX-PBI**, *λ*_ex_ = 520 nm. *c* = 1.0 × 10^−5^ M, 25 °C.

**Figure 8:**
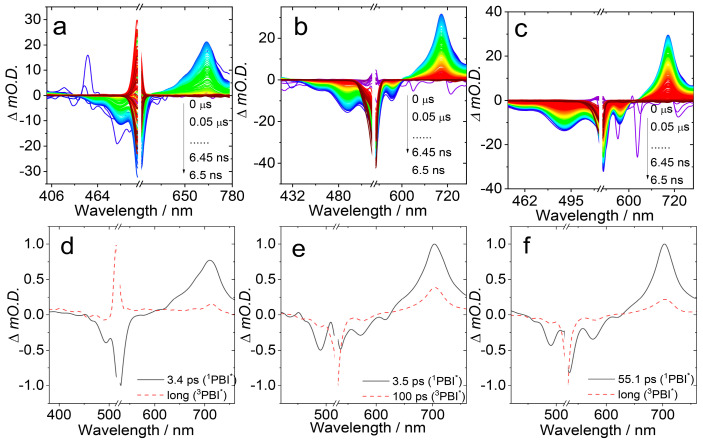
Femtosecond transient absorption spectra of (**a**) **DI-Rho-Et-PBI,** (**b**) **DI-Rho-HEX-PBI** and (**c**) **Rho-HEX-PBI**. Evolution associated difference spectra (EADS) obtained from global analysis of time resolved data in ACN for (**d**) **DI-Rho-Et-PBI**, (**e**) **DI-Rho-HEX-PBI** and (**f**) **Rho-HEX-PBI**, *λ*_ex_ = 520 nm. *c* = 1.0 × 10^−5^ M, 25 °C.

To investigate the ISC process, fs-TA spectroscopy measurements of **Rho-PBI** derivatives were taken in various solvents (Figure 7 and Figure 8 and S20). Figure 7 illustrates the fs-TA spectra of the Rho–PBI derivatives obtained in TOL within the 350–750 nm range. While the absorption and fluorescence spectra of **DI-Rho-Et-PBI** have been reported previously [44], other photophysical properties remain unexplored. In this study, we investigated the fs-TA spectra of **PBI-Br** in TOL and ACN and performed sequential global analysis to obtain the evolution-associated difference spectra (EADS) (Figure S20). Upon pulsed laser excitation, **PBI-Br** exhibits characteristic excited-state absorption (ESA) features in the range 350 ⟶ 600 nm, with peaks centered around 520 nm and 705 nm. This signal recovers to the baseline within 6.5 ns. Based on the sequential global analysis, the first species (represented by the black solid line) is assigned to the S_1_ state. Within 360 ps, the spectrum undergoes a slight red shift, corresponding to the formation of the T_1_ state, which possesses a significantly longer decay lifetime. A similar photophysical process was observed for **PBI-Br** in ACN, where no CS state was observed.

For **DI-Rho-Et-PBI**, characteristic ESA bands centered at 705 nm were observed, along with a negative absorption band near 520 nm (Figure 7a) [45]. This negative feature is attributed to the GSB band, as its spectral range overlaps with the steady-state UV–vis absorption spectra (Figure 1). The fs-TA spectra were analyzed using a global fitting method based on a sequential linear decay model (Figure 7). The first component, represented by the black line, corresponds to the Franck–Condon (FC) S_1_ state. With increasing delay time, an ISC process occurs over a timescale of 5.4 ps to generate the T_1_ state, which exhibits an absorption center at 520 nm. The structural features of the ESA band for this final species are in agreement with the ns-TA spectroscopy.

In ACN, the fs-TA spectra for **DI-Rho-Et-PBI** (Figure 8a) reveal an ESA band centered at 705 nm, with a GSB band at 520 nm. Global fitting analysis based on a three-component sequential decay model (Figure 8d) elucidates the excited-state evolution pathway: the initially populated Franck–Condon S_1_ state (represented by the black trace) undergoes ISC to the T_n_ state within 3.4 ps. Subsequently, the system further relaxes to the T_1_ state. Notably, the final species resulting from this transformation exhibits absorption at 520 nm, with a spectral profile that matches the triplet state ‘fingerprint’ observed in ns-TA spectral measurements. Although no signals of CS species were detected in the fs-TA spectra, electrochemical data suggest the possible existence of this transient species. We propose that for **DI-Rho-Et-PBI**, **DI-Rho-HEX-PBI**, and **Rho-HEX-PBI**, the S_1_ state undergoes ultrafast transition to a CS state (the instrument response function <120 fs). This CS state then rapidly evolves into T_n_ states or S_0_ state via CR; thus, no CS state was observed in the fs-TA spectra.

### 2.6. Theory Calculations

To evaluate the separation and orientation of the electron donor (Rho) and electron acceptor (PBI) in the dyad/triads, the ground state geometries were optimized using density functional theory (DFT) at the B3LYP/6-31G(d) level.

**Figure 9:**
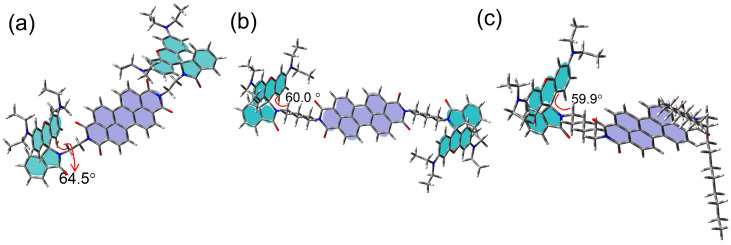
Optimized ground state geometry of (**a**) **DI-Rho-Et-PBI**, (**b**) **DI-Rho-HEX-PBI**, (**c**) **Rho-HEX-PBI** in gas phase. The selected dihedral angles are presented. Calculation was performed via DFT theory at B3LYP/6-31G(d) level with Gaussian 16.

As illustrated in Figure 9, the dihedral angle between the Rho and PBI planes in **DI-Rho-Et-PBI** was calculated to be 64.5°. With increasing linker length, the dihedral angles in **DI-Rho-HEX-PBI** and **Rho-HEX-PBI** slightly decreased to 60.0° and 59.9°, respectively. These subtle variations in dihedral angles suggest that the flexible chains effectively alleviate steric hindrance of the two moieties. This allows the electron donor and electron acceptor units to adopt a non-orthogonal alignment that is favorable for CS, as well as CR processes.

**Figure 10:**
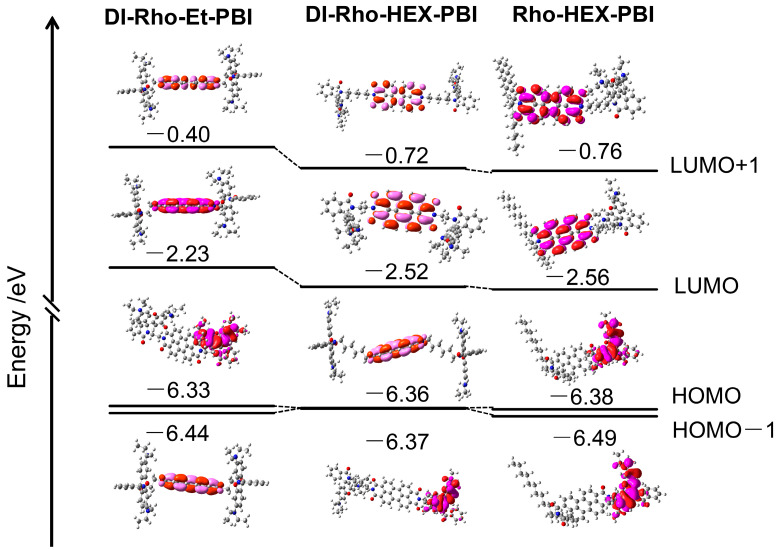
Selected frontier molecular orbitals of **DI-Rho-Et-PBI**, **DI-Rho-HEX-PBI** and **Rho-HEX-PBI**, based on the optimized ground state, in gas phase, respectively. Calculation was performed using DFT method at the CAM-B3LYP/6-31G(d) level with Gaussian 16.

Further, we performed DFT calculations to investigate the electron density distributions of the frontier molecular orbitals of the compounds (Figure 10). The highest occupied molecular orbitals (HOMO) of **DI-Rho-Et-PBI** and **Rho-HEX-PBI** are localized on the Rho unit, while the HOMO of **DI-Rho-HEX-PBI** is localized on the PBI unit. The lowest unoccupied molecular orbitals (LUMO) of all compounds are localized on the PBI unit. The HOMO and LUMO are clearly separated, indicating a negligible orbital overlap between the electron donor and electron acceptor. Consequently, the coupling between the electron donor and electron acceptor is weak, which supports the UV–vis absorption spectral results (Figure 1).

**Figure 11:**
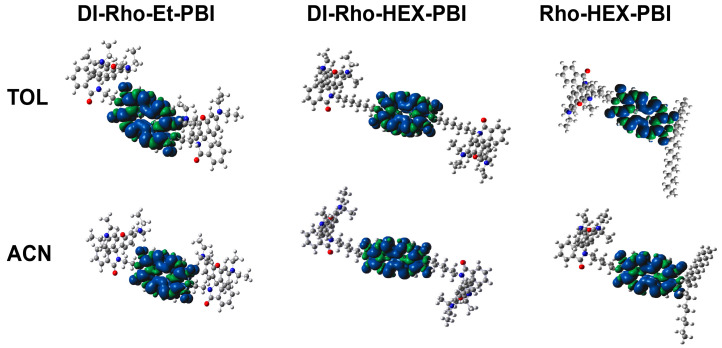
Spin density surfaces of the dyad/triads in the T_1_ state of **DI-Rho-Et-PBI**, **DI-Rho-HEX-PBI** and **Rho-HEX-PBI** in TOL and ACN, isovalue = 0.02. Calculations were performed with DFT theory at the CAM-B3LYP/6-31G(d) level with Gaussian 16.

Furthermore, the triplet state spin density distributions of the dyad in various solvents were investigated (Figure 11). For **DI-Rho-Et-PBI**, **DI-Rho-HEX-PBI**, and **Rho-HEX-PBI**, the spin densities in both TOL and ACN are confined on the PBI moiety, which is in agreement with the results observed in the ns-TA spectra.

The variations in *Φ*_Δ_ of the compounds can be rationalized by the spin–orbit coupling matrix elements (SOCMEs) of the ISC pathways (the SOCMEs were calculated with ORCA program) [46]. Taking the fluorescent PBI as a benchmark, its direct S_1_ to T_1_ ISC pathway exhibits a negligible SOCME value (<0.1 cm^−1^) and a substantial energy gap (Δ*E*_S1−T1_. Table S2). Computational results show that the SOCMEs for **DI-Rho-Et-PBI** and **Rho-HEX-PBI** increased to 1.11 cm^−1^ (S_1_ ⟶ T_4_) and 1.21 cm^−1^ (S_1_ ⟶ T_3_), respectively. This facilitates the ISC process. Furthermore, the reliability of this theoretical approach was validated by calculation of the SOCMEs of the ISC of benzophenone; a large SOCME was obtained using a similar computation method (S_1_ ⟶ T_2_, 21.09 cm^−1^), which is in the same order of magnitude as the literature reports (52.55 cm^−1^) [47]. Zero field splitting (ZFS) represents a critical physical property for characterizing the electronic structure of the lowest excited triplet state (T_1_). A previously reported planar PBI shows a ZFS *D* parameter of −0.0435 cm^−1^. In low–polarity toluene, the *D* values for **DI-Rho-Et-PBI**, **DI-Rho-HEX-PBI**, and **Rho-HEX-PBI** were calculated as 0.074, 0.085, and 0.072 cm^−1^, respectively. In solvent ACN, these *D* values decreased to 0.054, 0.060, and 0.053 cm^−1^, respectively (see Table 3).

**Scheme 2:**
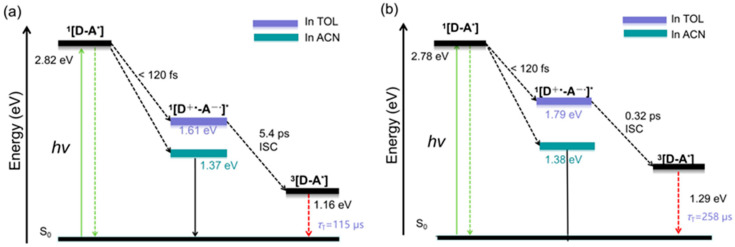
Simplified Jablonski diagram illustrating the photophysical processes involved in (**a**) **DI-Rho-Et-PBI** and (**b**) **DI-Rho-HEX-PBI**. The energy of the excited singlet states is derived from the spectroscopic data; the energy of CS states is obtained by electrochemical data; and the triplet state energy is calculated using TDDFT method at B3LYP/6-31G(d) level using Gaussian 16. The number of the superscript designates the spin multiplicity of the states. Please note the CS states are a mechanistic proposal, and they are not observed experimentally.

Based on the experiments, an energy diagram and the photophysical processes for the Rho-PBI triads are presented (Scheme 2). Upon photoexcitation of the PBI chromophore, the ^1^PBI* state is initially populated in both the dyad and triads. Transient kinetics reveal that for the **DI-Rho-Et-PBI** molecule in TOL, the ^1^PBI* state directly decays to the ^3^PBI* state within 5.4 ps. Neither FRET nor charge separation/charge recombination processes were observed with fs-TA spectra, despite the quenching of the fluorescence, which may be due to the ultrafast electron transfer or the energy transfer processes. Similar properties were observed in the polar solvent ACN. A similar trend was observed for the other two compounds.

## 3. Materials and Methods

### 3.1. Analytical Measurements

All chemicals used for the synthesis were of analytical grade and were used as received without purification. For synthesis, the reagents were analytically pure and used without further purification; the solvents are sufficient for spectroscopic studies, since we did not find any absorption/emission bands other than the compounds. UV–vis absorption spectra were obtained on a UV-2550 spectrophotometer (Shimadzu Ltd., Kyoto, Japan). Fluorescence spectra were recorded on FS5 spectrofluorometer (Edinburgh Instrument Ltd., Livingston, UK). Fluorescence quantum yields (*Φ*_F_) were measured with FS5 spectrofluorometer (Edinburgh Instrument Ltd., UK) based on relative method. The standard used is 2,6-diiodo-BODIPY (**2I-BDP**, *Φ*_F_ = 0.88 in toluene). The fluorescence quantum yield was calculated according to Equation (4). In this equation, *A* represents the absorbance at the excitation wavelength, *m* denotes the slope of the integrated fluorescence intensity versus absorbance plot, and *η* signifies the refractive index of the solvent. The subscripts “std” and “sam” refer to the standard reference and the test sample, respectively. The synthetic procedures and structural characterization data of target compounds, i.e., **DI-Rho-Et-PBI**, **DI-Rho-HEX-PBI** and **Rho-HEX-PBI**, are collected in the following sections. For other compounds, refer to the Supporting Information.

(4) $$\Phi_{F,sam}=\Phi_{F,std}\left(\frac{1-10^{-A_{std}}}{1-10^{-A_{sam}}}\right)\left(\frac{m_{sam}}{m_{std}}\right){\left(\frac{\eta_{sam}}{\eta_{std}}\right)}^{2}$$

### 3.2. Nanosecond Transient Absorption Spectra

The ns-TA spectra were recorded on LP980 laser flash photolysis spectrometer (Edinburgh Instruments, Ltd., UK). Collinear configuration of the pump and probe beams was used for the measurements. The sample solution was purged with N_2_ for 15 min and sealed for measurement. The samples were excited with nanosecond pulsed laser (Surelite I-10, Continuum, San Jose, CA, USA; the wavelength is tunable in the range of 400–2400 nm). The typical laser energy is ca. 10 mJ per pulse. The signal was digitized with a Tektronix TDS 3012B oscilloscope (Beaverton, OR, USA). The data were analyzed with L900 software. By comparing the UV–vis absorption spectra before and after the measurements, the spectral features remained unchanged, thereby ruling out any potential laser-induced photodegradation.

### 3.3. Femtosecond Transient Absorption Spectroscopy

Femtosecond transient absorption spectra (fs-TA) were acquired on a system based on a Ti/sapphire regenerative amplifier (Amplitude Pulsar, Pessac, France) pumped by a home-built Ti/sapphire oscillator. The system produces 40 fs pulses at 800 nm, with a repetition rate of 1 kHz and an average power of 450–500 mW. The instrument time resolution is 150 fs. Excitation pulses at 340 nm were produced by third harmonic generation from the output of a commercial parametric amplifier (TOPAS, Light Conversion, Vilnius, Lithuania) using two BBO crystals. The polarization of the pump beam was set at the magic angle relative to the probe beam to eliminate anisotropic decay. The probe beam was generated by focusing a fraction of the fundamental 800 nm laser pulse onto a rotating 3 mm CaF_2_ nonlinear optical crystal to produce broadband white-light continuum while preventing optical damage. A motorized linear translation stage introduced precise pump probe temporal delays in the excitation beam path. After spatial and temporal overlap at the sample plane, the dispersed probe spectrum was analyzed using a high-throughput imaging spectrograph coupled to a CCD detector. We avoided repeated excitation of the same sample volume by translating the entire quartz flow cell (2 mm pathlength) mounted on a motorized stage. The translation followed a Lissajous figure pattern (2:3 ratio), implemented via the instrument’s control software (fs-TA system, HARPIA Service APP 1.7, Light Conversion, Lithuania). This pattern continuously moves the cell so that the pump beam illuminates different spatial positions over time, effectively refreshing the irradiated sample volume. Consequently, the same volume is never excited by two consecutive pulses, effectively eliminating photobleaching and accumulation of long-lived triplet states. The transient absorption data were analyzed using Glotaran 1.5.1 software suite [48]. First, singular-value decomposition (SVD) was performed to determine the optimal number of kinetic components for subsequent global analysis. All datasets were subjected to chirp correction prior to global fitting with the TIMP algorithm.

### 3.4. Density Functional Theory (DFT) Calculations

DFT calculations were performed by using the Gaussian 16 package [49]. The ground state geometries were optimized using DFT theory with the CAM-B3LYP functional and the 6-31G(d) basis set. The excitation energies of the compounds were calculated using TD-DFT at CAM-B3LYP/6-31G(d) level. Spin–orbit coupling matrix elements (SOCMEs) were calculated using the Spin–Orbit Mean-Field (SOMF) approximation, as implemented in ORCA 5.0 [50,51]. The excited-state wavefunctions were obtained via TD-DFT at the CAM-B3LYP/def2-TZVP level, where the lowest 10 singlet and 10 triplet roots were solved to ensure an adequate state-mixing manifold. The coupling was evaluated within the quasi-degenerate perturbation theory (QDPT) framework.

### 3.5. Synthesis of Compounds **2** and **3**

Rhodamine B (1.0 g, 2.0 mmol) was dissolved in methanol (20 mL). Under stirring at room temperature, ethylenediamine (1.0 mL, 15.1 mmol) was added dropwise over a period of 30 min. The resulting mixture was refluxed for 24 h. After the reaction, the solvent was removed under reduced pressure to give an orange residual. The crude product was then dissolved in 1 M hydrochloric acid (HCl, 100 mL), forming a clear-red solution. Subsequently, 1 M sodium hydroxide (NaOH, 120 mL) was slowly added to the aqueous solution with stirring to adjust the pH to 8–9, resulting in a pink precipitate. The final product was dried under vacuum for 24 h to afford intermediate **2** (0.9 g), yield 90%. ^1^H NMR (400 MHz, CDCl_3_): *δ* 7.88–7.89 (m, 1H), 7.39–7.45 (m, 2H), 7.05–7.08 (m, 1H), 6.23–6.42 (m, 6H), 3.31 (q, 8H, *J* = 6.8 Hz), 3.17 (t, 2H, *J* = 6.8 Hz), 2.36 (t, 2H, *J* = 6.7 Hz), 1.18–1.15 (m, br, 14H), MALDI-HRMS([C_30_H_36_N_4_O_2_]^+^): calcd *m/z* = 485.2911, found *m/z* = 485.2925.

Compound **3** was synthesized following similar procedures, and a pink solid was obtained (1.1 g, 88% yield). ^1^H NMR (400 MHz, CDCl_3_): *δ* 7.87 (s, 1H), 7.42 (s, 2H), 7.06–7.08 (m, 1H), 6.35–6.43 (m, 4H), 6.26 (s, 2H), 3.34 (q, 8H, *J* = 20 Hz), 3.09 (t, 2H, *J* = 12 Hz), 2.95 (t, 2H, *J* = 16 Hz), 2.85 (s, 1H), 1.59–1.71 (m, 2H), 1.09–1.17 (m, 19H). MALDI-HRMS([C_34_H_44_N_4_O_2_]^+^): calcd *m/z* = 541.3537, found *m/z* = 541.3544.

Synthesis of the compound **DI-Rho-Et-PBI**. Compound **1** (1.0 g, 2.56 mmol), 2 (3.3 g, 6.68 mmol), and Et_3_N (0.8 g, 7.78 mmol) were dissolved in *N*,*N*-dimethylformamide (DMF) (60 mL). The mixture was heated at 160 °C for 12 h. After cooling to room temperature, the mixture was poured into water and extracted with dichloromethane (DCM) solvent (3 × 30 mL). The combined organic layers were dried over anhydrous Na_2_SO_4_, and the solvent was evaporated under reduced pressure. The crude product was purified by column chromatography (DCM/MeOH = 20:1, *v*/*v*) to afford 0.7 g (yield: 20%) of a red solid. M.p.: >250 °C. ^1^H NMR (400 MHz, CDCl_3_) *δ* 8.59–8.48 (m, 8H), 7.90 (s, 2H), 7.41 (t, 4H, *J* = 3.5 Hz), 7.02 (d, 2H, *J* = 5.8 Hz), 6.51–6.46 (m, 4H), 6.38 (s, 4H), 6.27 (d, 4H, *J* = 10.9 Hz), 4.08 (t, 4H, *J* = 12 Hz), 3.35–3.30, (m, 16H), 3.1 (t, 4H, *J* = 12 Hz), 1.17–1.13 (m, br, 24H). ^13^C NMR (100 MHz, CDCl_3_): δ 167.0, 161.5, 151.5, 146.5, 132.4, 129.2, 126.0, 121.7, 120.8, 106.0, 103.9, 95.8, 75.3, 63.4, 51.5, 42.2, 37.5, 27.8, 10.7. MALDI-HRMS([C_84_H_76_N_8_O_8_]): calcd *m*/*z* = 1324.5800, found *m*/*z* = 1324.5830. The synthetic procedures for other compounds are presented in the Supporting Information.

## 4. Conclusions

In summary, we prepared a series of electron donor–acceptor dyads and triads with perylenebisimide (PBI) as the electron acceptor and closed-ring rhodamine (Rho) as the electron donor. The Rho-PBI dyad/triads contain flexible alkyl linkers (ethylene or hexylene units). Steady-state absorption spectra indicate that the electronic interaction between the electron donor and electron acceptor units is negligible at ground state. Nevertheless, fluorescence measurements reveal that the emission of the PBI unit is strongly quenched, even in non-polar solvents such as toluene. This behavior is attributed to the photo-induced electron transfer (PET) process between the two chromophores. Furthermore, a Förster resonance energy transfer (FRET) process between PBI (energy donor) and open-ring rhodamine (energy acceptor) may also exist. Femtosecond transient absorption spectra did not show electron transfer, probably due to the ultrafast charge recombination to the localized triplet state (^3^PBI) and the ground state (S_0_ state). Nanosecond transient absorption measurements confirm the formation of the PBI triplet excited state (lifetime is up to 126 μs). The triplet quantum yields of the dyad and triads are low (<3%). The mutual orientation of the electron donor and acceptor is not fixed; therefore, no efficient spin–orbit charge recombination-induced ISC (SOCT-ISC) exists. This study provides new insight into the photophysical behavior of flexible D-A systems and offers a useful reference for the molecular design of heavy-atom-free triplet photosensitizers.

## Figures and Tables

**Table 1:** Photophysical data of the compounds ^1^.

Compounds	*λ*_abs_ ^2^ (*ε* ^3^)	*λ*_F_ ^4^	*Φ*_F_ ^5^	*Φ*_Δ_ ^6^
**DI-Rho-Et-PBI**	527 (5.9)	534	0.2	2.9
**DI-Rho-HEX-PBI**	524 (5.3)	535	1.7	2.1
**Rho-HEX-PBI**	526 (5.8)	537	5.9	1.4
**PBI-Br**	524 (0.8)	533	95.2	7.8
**Rho-Pr**	314 (0.9)	- ^7^	- ^7^	- ^7^

^1^ In TOL (*c* = 1.0 × 10^−5^ M). For all the experimental results, the determination error is less than 5%. ^2^ Absorption wavelength, in nm, *c* = 1.0 × 10^−5^ M. ^3^ Molar absorption coefficient. *ε* values are in 10^4^ M^−1^ cm^−1^. ^4^ Fluorescence emission maxima wavelength, in nm. ^5^ Absolute Fluorescence quantum yield. ^6^ Singlet oxygen quantum yield, 2,6-diiodoBodipy (**2I-BDP**) was used as standard compound (*Φ*_Δ_ = 87% in DCM), *λ*_ex_ = 520 nm. ^7^ Not observed.

**Table 2:** Redox potential, CS free energy (Δ*G*_CS_) ^1^ and CS energy states (*E*_CS_) for compounds in DCM.

Compounds	*E*_RED_/V ^2^	*E_ox_*/V ^2^	Δ*G*_CS_/eV				*E*_CS_/eV			
			n-HEX	TOL	DCM	ACN	n-HEX	TOL	DCM	ACN
**DI** **-** **Rho** **-** **Et** **-** **PBI**	−1.27, −1.08	0.51, 0.73	−0.66	−0.73	−0.92	−0.97	1.68	1.61	1.42	1.37
**DI** **-** **Rho** **-** **HEX** **-** **PBI**	−1.26, −1.06	0.54, 0.71	−0.43	−0.55	−0.87	−0.96	1.91	1.79	1.47	1.38
**Rho** **-** **HEX** **-** **PBI**	−1.31, −1.11	0.47, 0.64	−0.43	−0.54	−0.87	−0.96	1.92	1.80	1.47	1.38
**PBI** **-** **Br**	−1.27, −1.08	- ^3^	- ^4^	- ^4^	- ^4^	- ^4^	- ^4^	- ^4^	- ^4^	- ^4^
**Rho** **-** **Pr**	- ^3^	0.57, 0.70	- ^4^	- ^4^	- ^4^	- ^4^	- ^4^	- ^4^	- ^4^	- ^4^

^1^ *E*_00_ = 2.34 eV. *E*_00_ = 1240/*λ*. *E*_00_ is energy level for the singlet excited state, *λ* is the wavelength of the crossing point of normalized UV–vis absorption spectra and fluorescence emission spectra. ^2^ The value is obtained by setting the oxidation wave of Fc^+^/Fc as 0. ^3^ Not observed. ^4^ Not applicable. For all the experimental results, the determination error is less than 5%.

**Table 3:** Zero field splitting parameters (*D* and *E*), spin–spin and spin–orbit (cm^−1^) of the compounds in TOL calculated at the optimized ground state ^1^.

Compounds	ZFS		Spin–Spin		Spin–Orbit	
	*D*/cm^−1^	*E*/cm^−1^	*D*/cm^−1^	*E*/cm^−1^	*D*/cm^−1^	*E*/cm^−1^
**DI-Rho-Et-PBI**	0.074	0.013	−0.004	−0.014	0.077	0.027
**DI-Rho-HEX-PBI**	0.085	0.016	−0.004	−0.014	0.088	0.030
**Rho-HEX-PBI**	0.072	0.014	−0.004	−0.016	0.076	0.029

^1^ The calculations of the zero field splitting parameters (ZFS), spin–spin and spin–orbit were performed at CAM-B3LYP/EPR-II level (toluene) using the ORCA 6.0 programs [46].

## Data Availability

The original contributions presented in this study are included in the article and Supplementary Materials. Further inquiries can be directed to the corresponding author.

## Supplementary Materials

The following supporting information can be downloaded at: https://www.mdpi.com/article/10.3390/molecules31111859/s1.
