# Supplementary material for: Preparation and Photophysical Study of Rhodamine–Perylenebisimide Electron Donor–Acceptor Dyad/Triads Containing Flexible Linkers
Source: Molecules. 2026 May 28;31(11):1859. doi: 10.3390/molecules31111859 (PMC13258424; doi:10.3390/molecules31111859)
Supplement: Supplementary file 1 [file molecules-31-01859-s001.zip › molecules-4266317-supplementary.pdf]

Supporting Information for:

Preparation and Photophysical Study of Rhodamine-Perylenebisimide Electron Donor-Acceptor Dyad/Triads Containing Flexible Linkers

Xin Guan [a], Haotian Bai [b], Jianzhang Zhao [a,*], Yan Wan [b,*]

[a] State Key Laboratory of Fine Chemicals, Frontiers Science Center for Smart Materials Oriented Chemical Engineering, School of Chemical Engineering, Dalian University of Technology, Dalian 116024, P. R. China. *Email: zhaojzh@dlut.edu.cn

[b] College of Chemistry, Beijing Normal University, Beijing 100875, P. R. China. E-mail: wanyan@bnu.edu.cn

Contents

1. Syntheses of Compounds. ········ S3
2. NMR and HRMS Spectra ········ S5
3. UV–vis Absorption Spectra ········ S14
4. Fluorescence Spectra ········ S14
5. Electrochemistry Study ········ S15
6. Nanosecond Transient Absorption Spectra ········ S15
7. Femtosecond Transient Absorption Spectra ········ S15
8. Photophysical Data of the Compounds ········ S18
9. Theoretical Calculations ········ S19

1. Syntheses of Compounds

Synthesis of Compound DI-Rho-HEX-PBI. Compound **1** (0.5 g, 1.28 mmol), **3** (1.6 g, 3.11 mmol), and Et_3N (0.4 g, 3.89 mmol) were dissolved in DMF (30 mL), the mixture was heated at 160 °C for 9.5 h. Upon completion, the reaction mixture was cooled to room temperature, and the solvent was removed under reduced pressure. The resulting red-black solid was washed thoroughly with water (200 mL), and dried in a vacuum oven at 60 ℃. Purification via column chromatography (DCM/MeOH = 500:1, v/v) afford 0.4 g (yield: 23%) of a purple solid. M.p.: >250 °C. ^1H NMR (400 MHz, $CDCl_3$,) δ 8.58 (d, 4H, J = 7.9 Hz), 8.49 (d, 4H, J = 8.0 Hz), 7.89 – 7.86 (m, 2H), 7.42 – 7.40 (m, 4H), 7.08 – 7.06 (m, 2H), 6.45 (s, 2H), 6.43 (s, 2H), 6.39 (d, 4H, J = 2.1 Hz), 6.29 – 6.26 (m, 4H), 4.08 (t, 4H, J = 7.0 Hz), 3.35 – 3.30 (m, 16H), 3.10 (t, 4H, J = 5.9 Hz), 1.13-1.17 (m, br, 40H). ^{13}C NMR (100 MHz, $CDCl_3$): δ 168.0, 163.5, 163.0, 153.6, 148.7, 134.0, 132.1, 131.5, 131.1, 130.1, 129.1, 129.0, 127.9, 125.9, 123.7, 123.2, 122.8, 108.0, 106.0, 97.7, 77.2, 77.0, 76.8, 64.9, 44.7, 44.4, 40.4, 36.6, 31.0, 30.1, 29.3, 28.2, 27.1, 26.7, 26.5, 22.7, 14.1, 12.6. MALDI-HRMS([$C_{92}H_{92}N_8O_8$]): calcd m/z = 1436.7960, found m/z = 1436.7050.

Synthesis of Compound Rho-HEX-PBI. Compound **4** (0.2 g, 0.35 mmol), **3** (0.3 g, 0.58 mmol), and Et_3N (0.04 g, 0.39 mmol) were dissolved in DMF (16 mL), the mixture was heated at 160 ℃ for 7 h. On completion of the reaction, the solvent was collected by filtration and washed with saturated DCM solvent (3 × 20 mL) and the organic layer was dried over Na_2SO_4, the solvent was evaporated under reduced pressure. The crude product was purified by column chromatography (silica gel; DCM/MeOH = 200:1, v/v), The compound **Rho-HEX-PBI** was obtained as a red powder (38.8 mg, yield: 10%). M.p.: ＞250 °C. ^1H NMR (400 MHz, $CDCl_3$) δ 8.67 – 8.62 (t, 4H, J = 8.0 Hz), 8.59 – 8.57 (d, 4H, J = 6.1 Hz), 7.43 (s, 3H), 7.05 (s, 1H), 6.45 – 6.28 (m, 6H), 4.15 – 4.08 (m, 4H), 3.33 (s, 10H), 1.25–1.21 (m, br, 53H), 0.84 (s, 6H). ^{13}C NMR (100 MHz, $CDCl_3$,): δ 168.0, 163.0, 153.6, 153.3, 148.7, 134.2, 132.1, 131.5, 129.0, 127.9, 123.7, 122.8, 122.7, 108.0, 106.0, 97.7, 77.2, 77.0,

76.8, 64.9, 46.2, 40.4, 28.2, 27.1, 26.7, 12.6. MALDI-HRMS($[C_{78}H_{91}N_5O_6]^-$): calcd *m/z* = 1194.6150, found *m/z* = 1193.6973.

2. NMR and HRMS Spectra of compounds

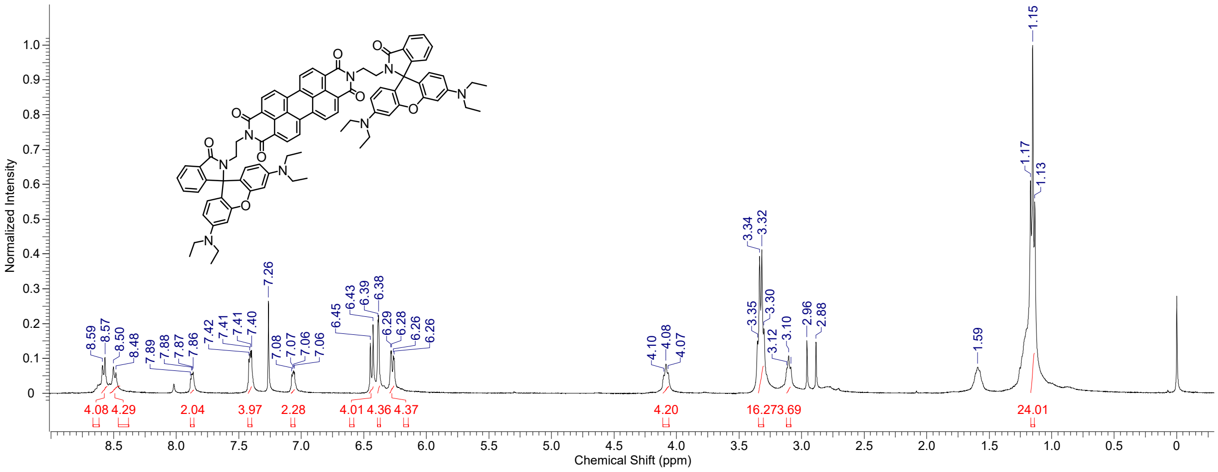

Figure S1. ^{1}H NMR spectrum of **DI-Rho-Et-PBI** (400 MHz, $CDCl_3$), 25 °C.

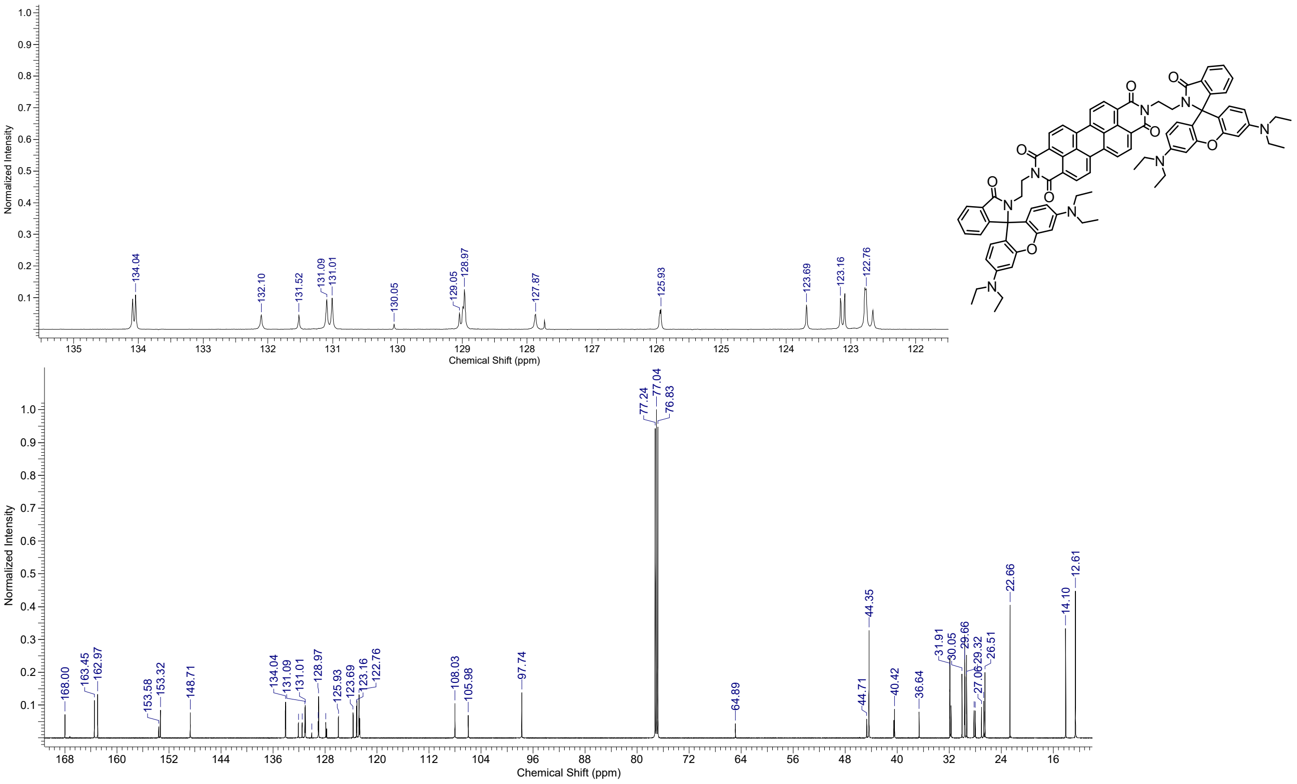

Figure S2. ^{13}C NMR spectrum of compound **DI-Rho-Et-PBI** (126 MHz, $CDCl_3$), 25 °C.

Compound	Formula	Exact Mass	Mol.Wt	m/z
DI-Rho-Et-PBI	$C_{84}H_{76}N_8O_8$	1324.5786	1325.5800	1324.5786<br>（100.0%）<br>1325.5820

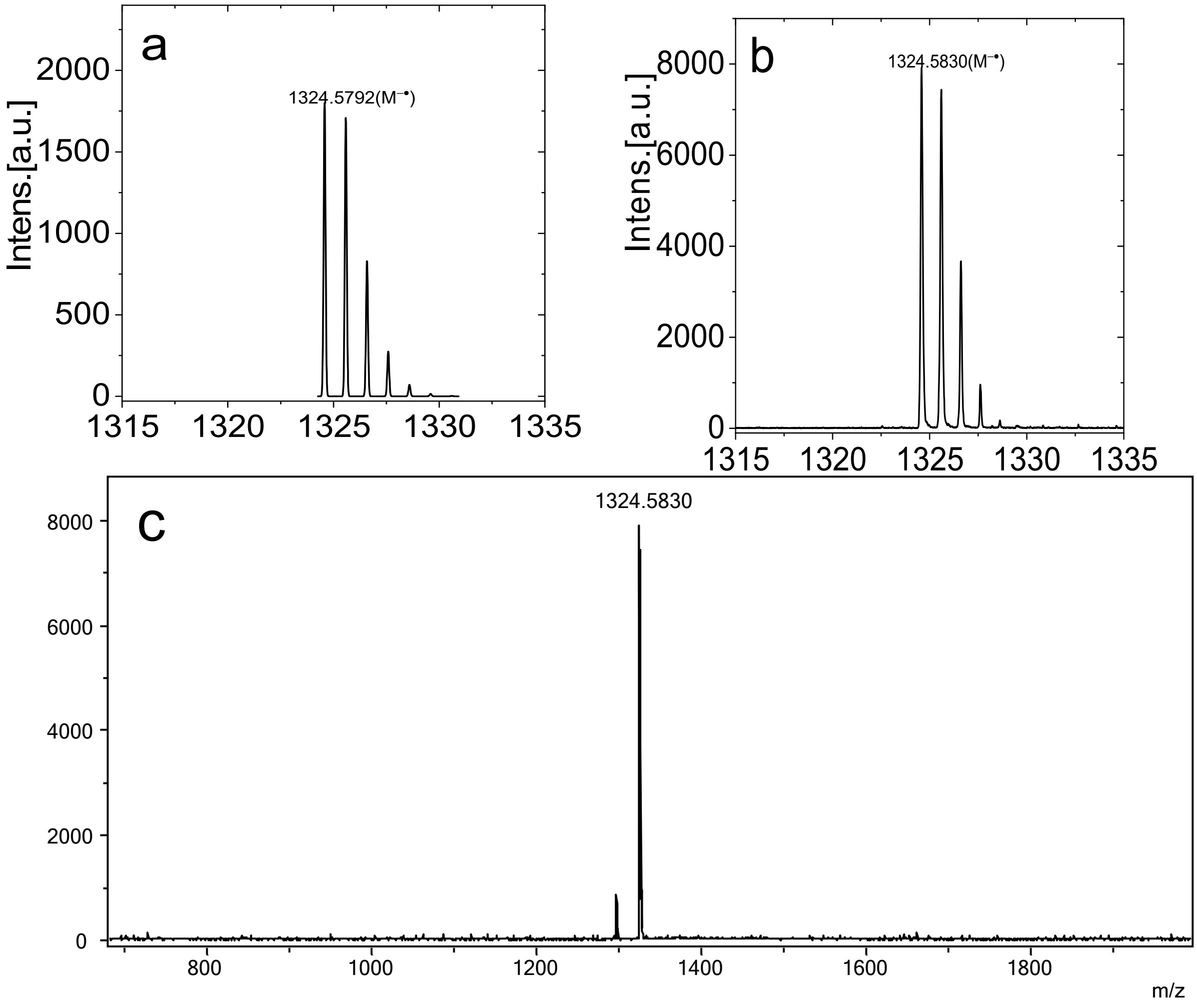

Figure S3. (a) ChemDraw predicted mass spectrum of **DI-Rho-Et-PBI**. (b) Magnified presentation of the molecular ion peak in the mass spectrum of **DI-Rho-Et-PBI** (c) MALDI HR MS of compound **DI-Rho-Et-PBI**. 25 °C.

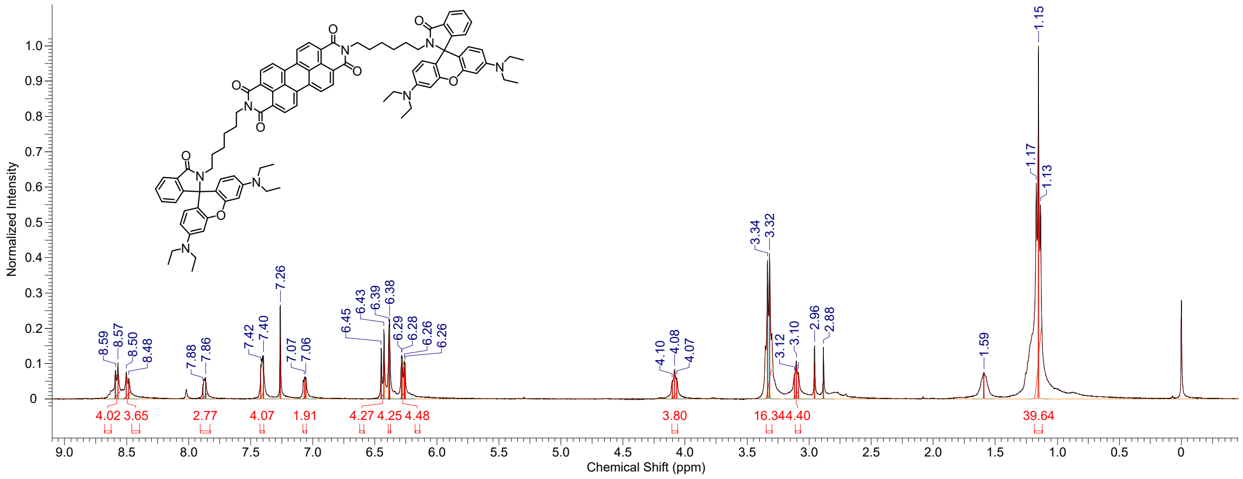

Figure S4. ^{1}H NMR spectrum of **DI-Rho-HEX-PBI** (400 MHz, $CDCl_3$), 25 °C.

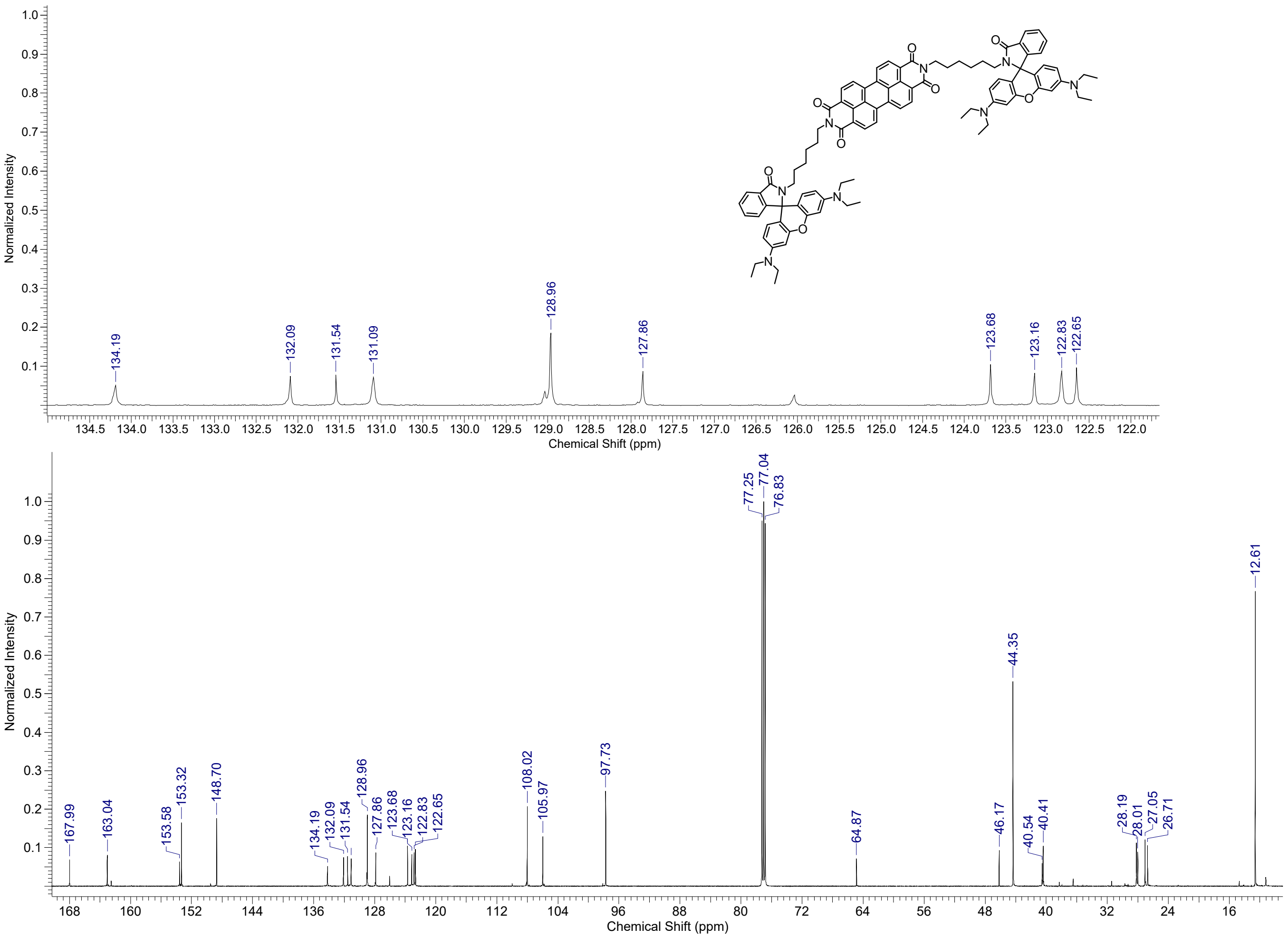

Figure S5. ^{13}C NMR spectrum of compound **DI-Rho-HEX-PBI** (126 MHz, $CDCl_3$), 25 °C.

Compound	Formula	Exact Mass	Mol.Wt	m/z
DI-Rho-HEX-PBI	$C_{92}H_{92}N_8O_8$	1436.7038	1437.7960	1436.7038<br>（100.0%）<br>1437.7072

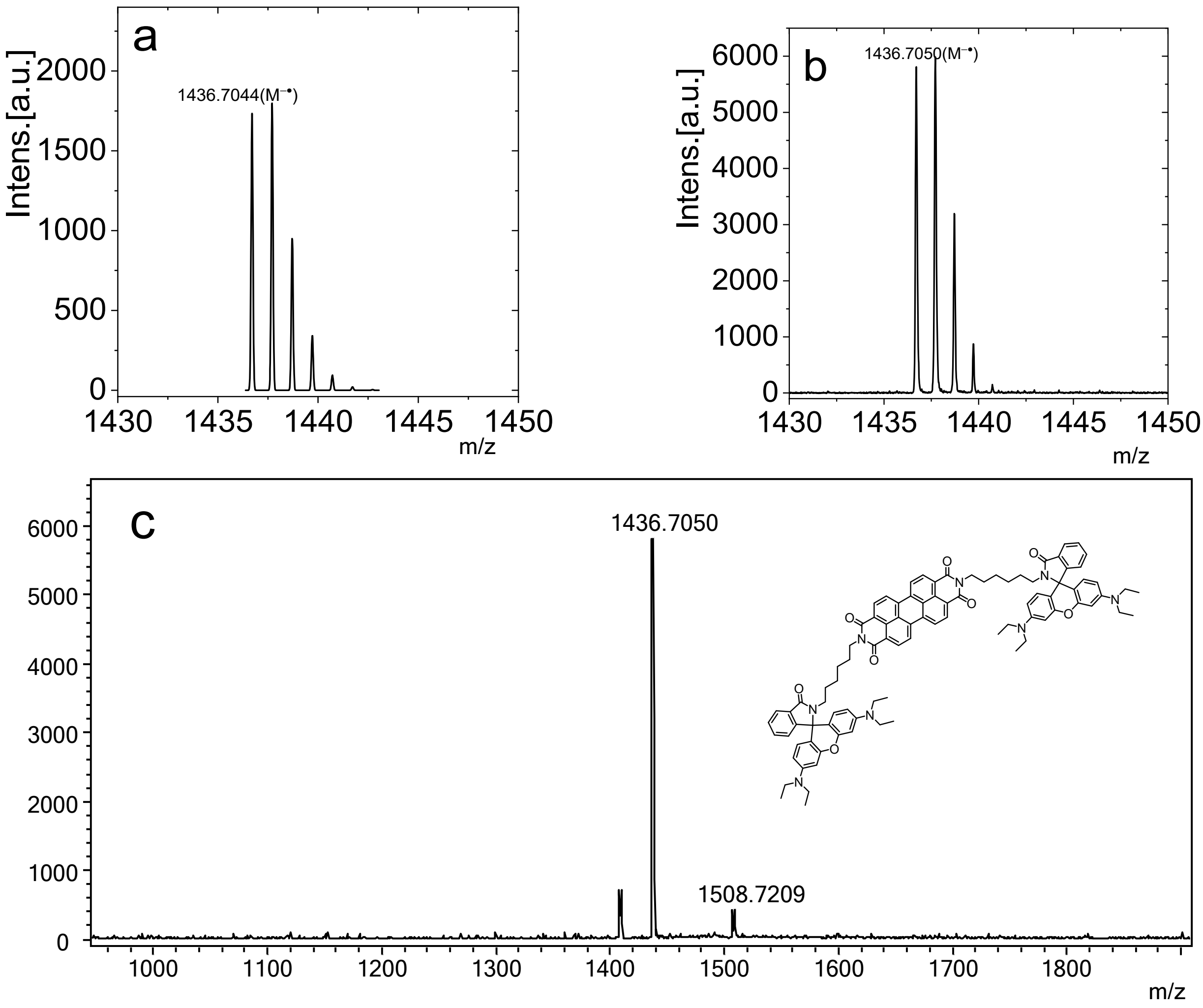

Figure S6. (a) ChemDraw predicted mass spectrum of **DI-Rho-HEX-PBI**. (b) Magnified presentation of the molecular ion peak in the mass spectrum of **DI-Rho-HEX-PBI** (c) MALDI HR MS of compound **DI-Rho-HEX-PBI**. 25 °C.

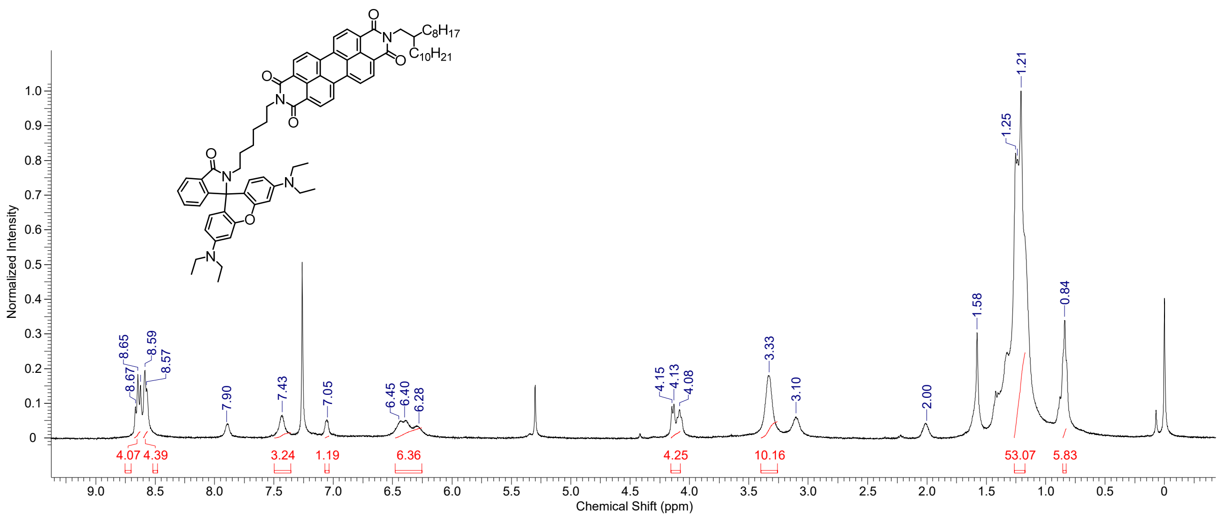

Figure S7. ^{1}H NMR spectrum of **Rho-HEX-PBI** (400 MHz, $CDCl_3$), 25 °C.

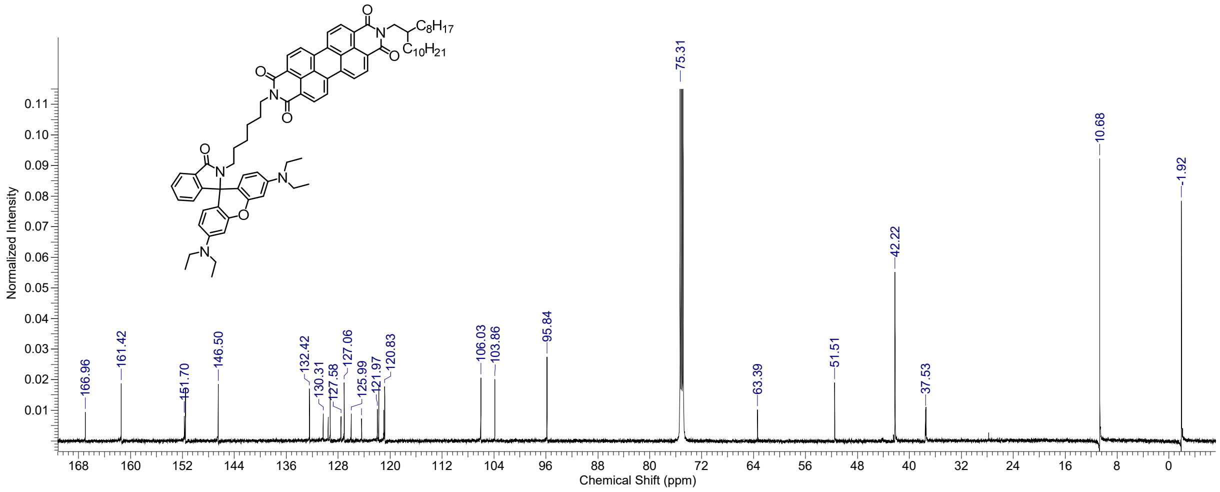

Figure S8. ^{13}C NMR spectrum of compound **Rho-HEX-PBI** (126 MHz, $CDCl_3$), 25 °C.

Compound	Formula	Exact Mass	Mol.Wt	m/z
Rho-HEX-PBI	$C_{78}H_{91}N_5O_6$	1193.6969	1194.6150	1193.6969<br>（100.0%）<br>1194.7003

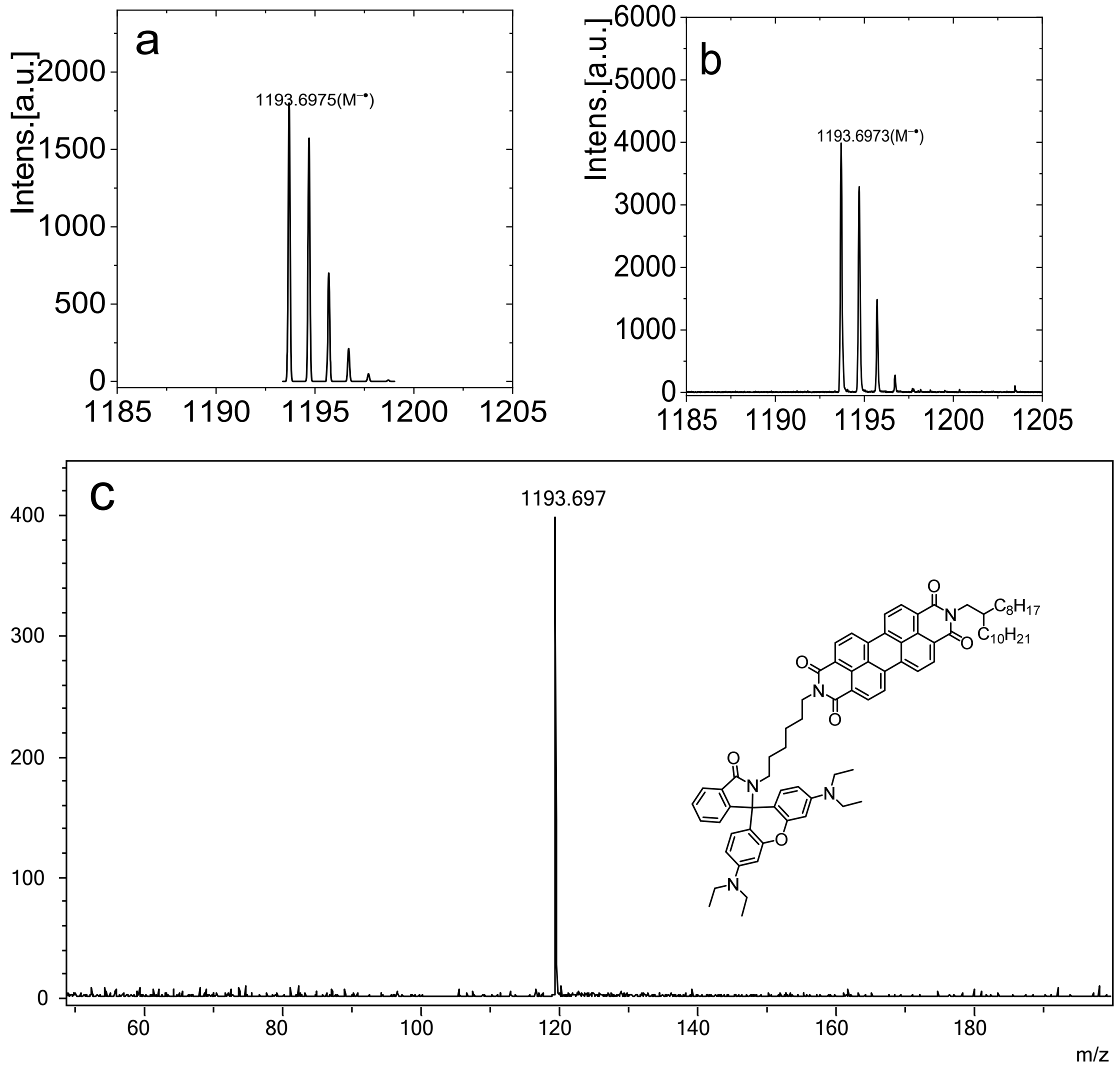

Figure S9. (a) ChemDraw predicted mass spectrum of **Rho-HEX-PBI**. (b) Magnified presentation of the molecular ion peak in the mass spectrum of **Rho-HEX-PBI** (c) MALDI HR MS of compound **Rho-HEX-PBI**. 25 °C.

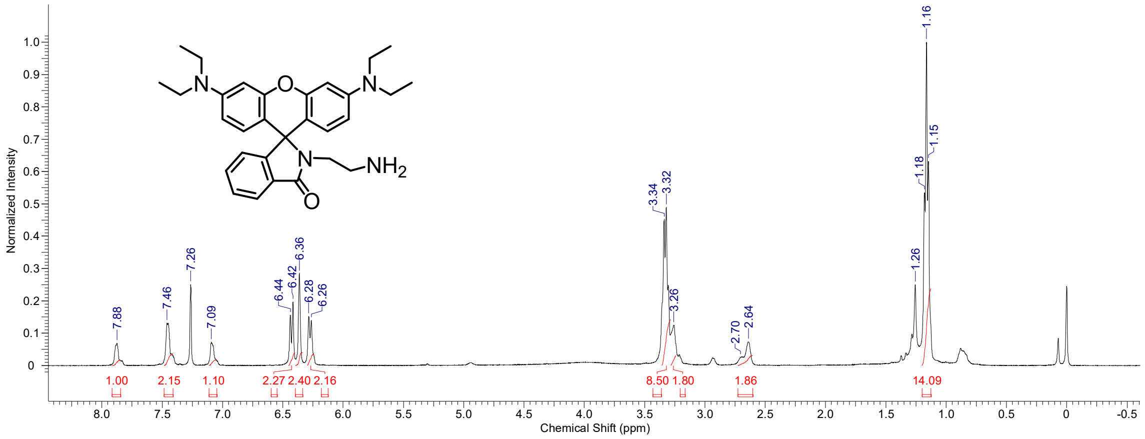

Figure S10. ^{1}H NMR spectrum of **2** (400 MHz, $CDCl_3$), 25 °C.

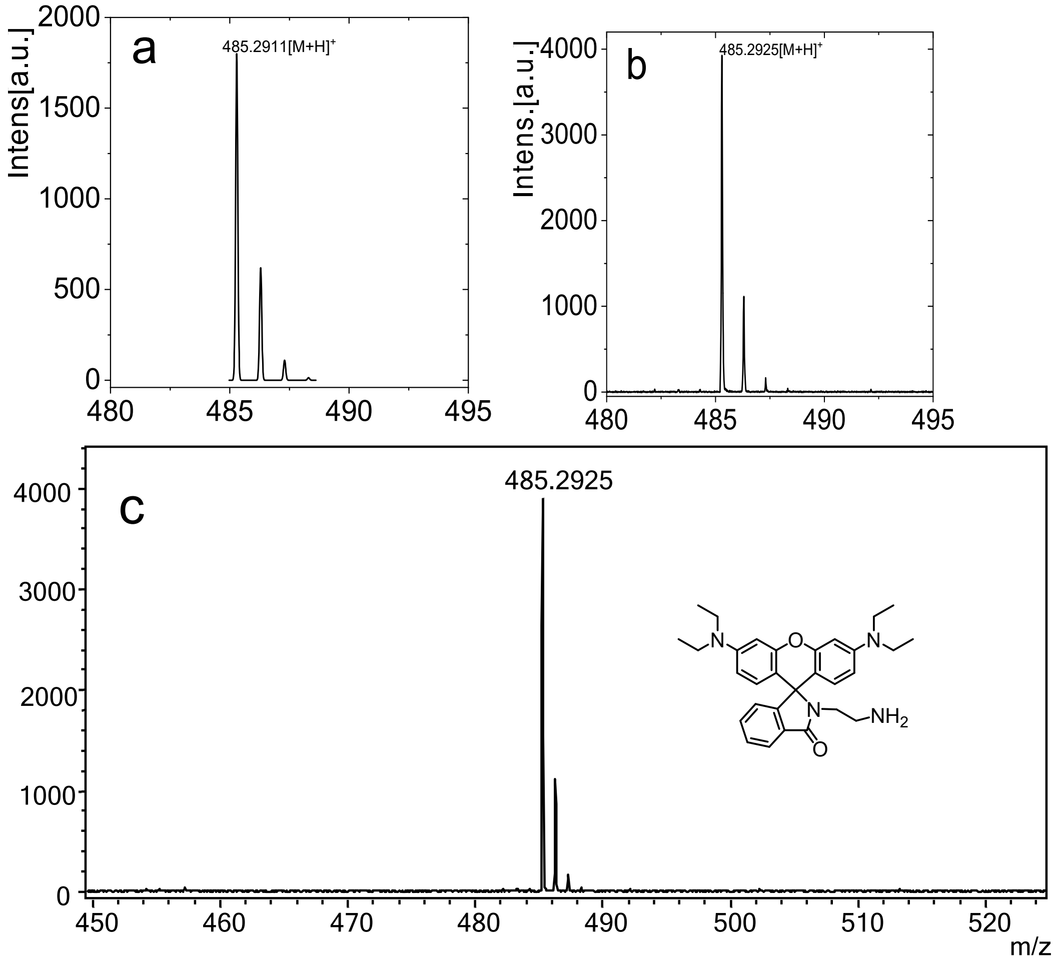

Figure S11. (a) ChemDraw predicted mass spectrum of Compound **2**. (b) Magnified presentation of the molecular ion peak in the mass spectrum of Compound **2** (c) MALDI HR MS of compound **2**. 25 °C.

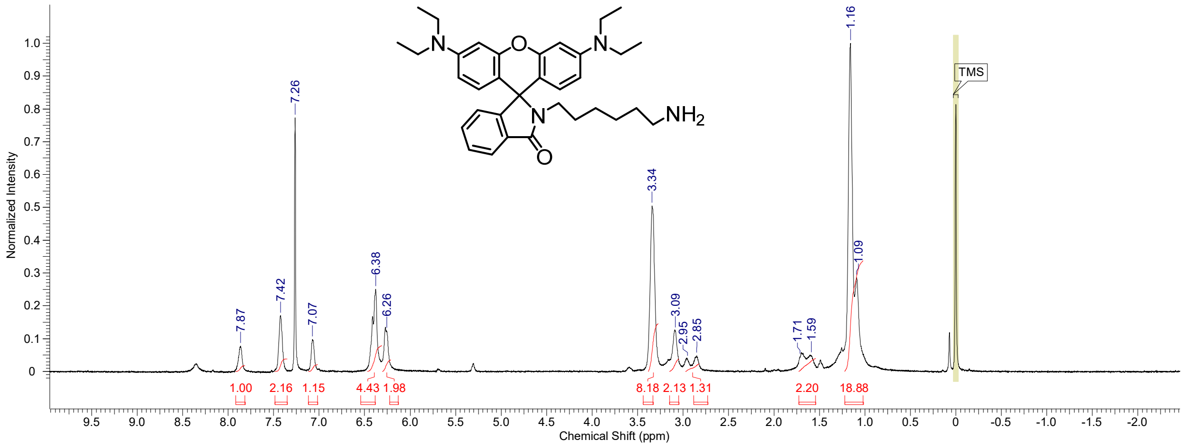

Figure S12. ^{1}H NMR spectrum of **3** (400 MHz, $CDCl_3$), 25 °C.

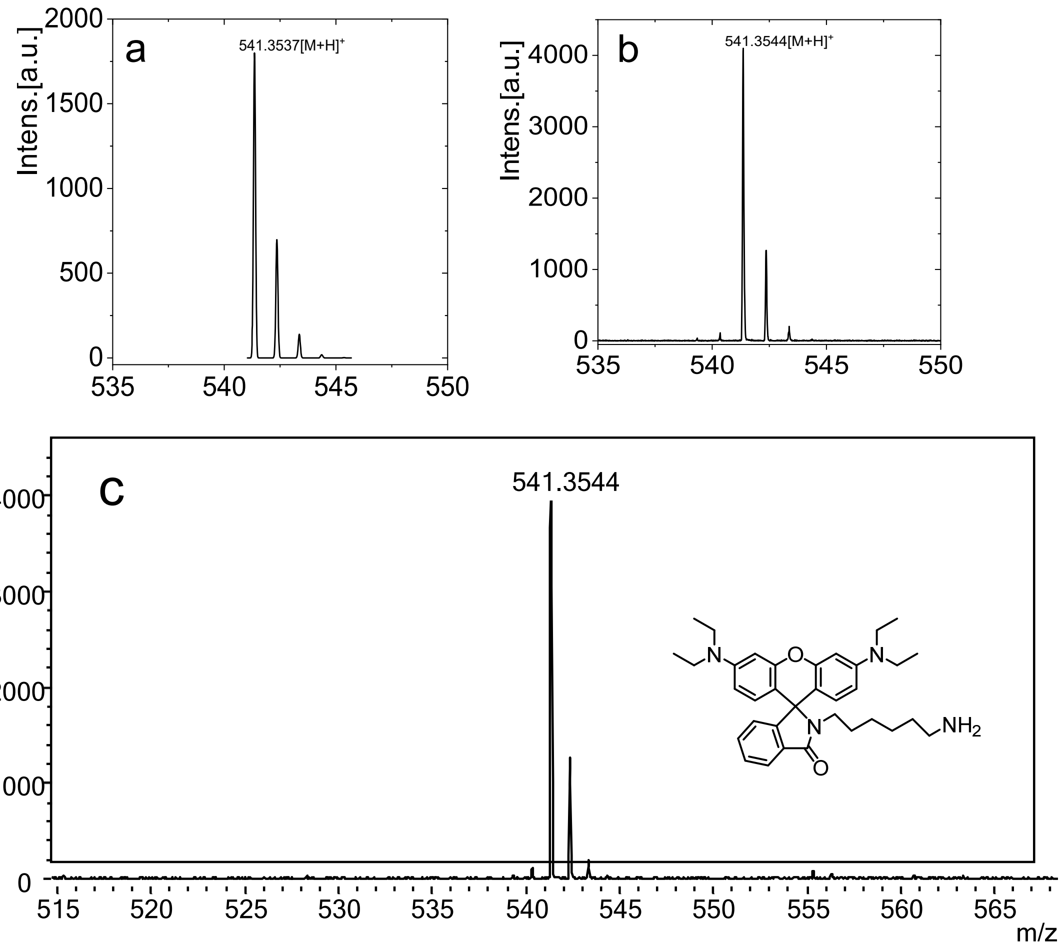

Figure S13. (a) ChemDraw predicted mass spectrum of Compound **3**. (b) Magnified presentation of the molecular ion peak in the mass spectrum of Compound **3** (c) MALDI HR MS of compound **3**. 25 °C.

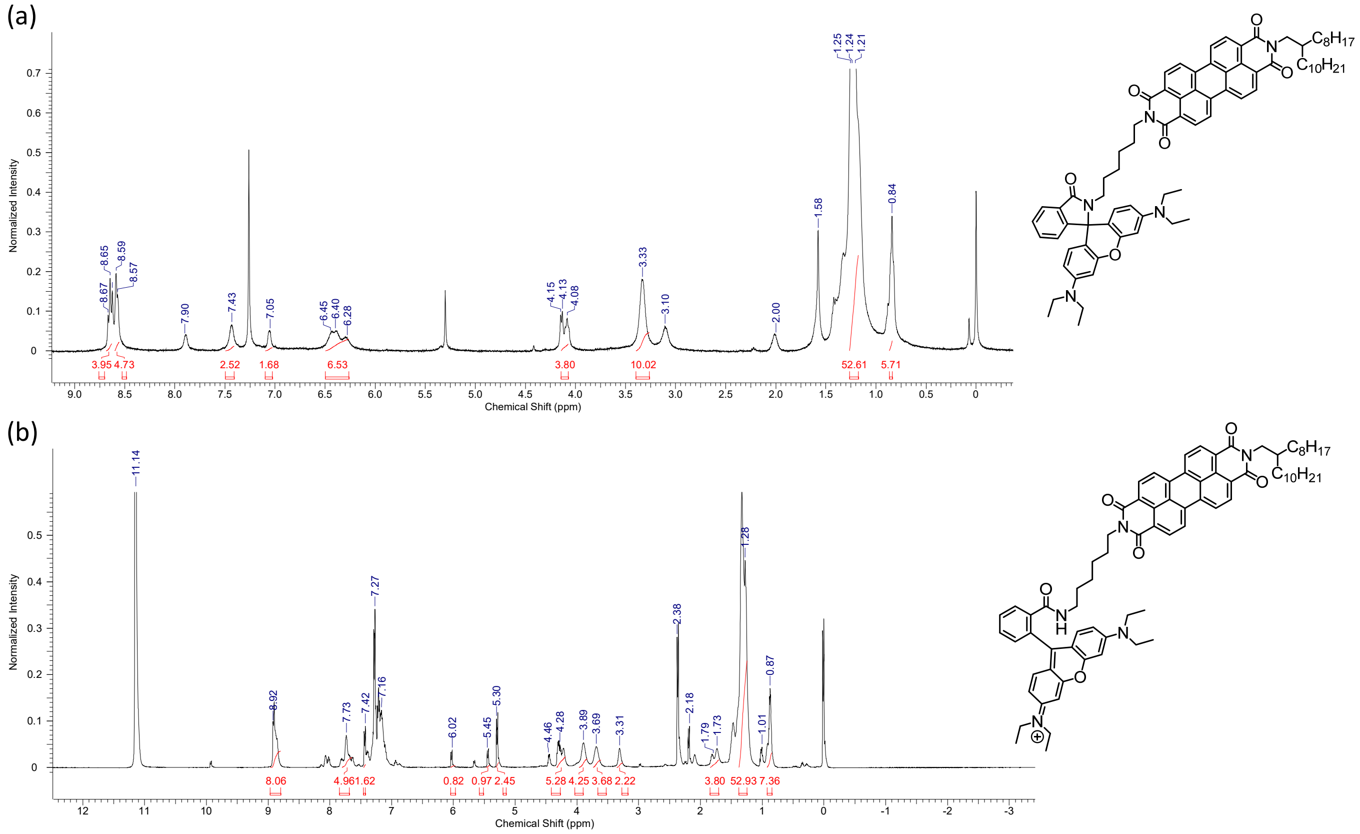

Figure S14. ^{1}H NMR spectra of **Rho-HEX-PBI** in the (a) absence and (b) presence of TFA-d, (400 MHz, $CDCl_3$), 25 °C.

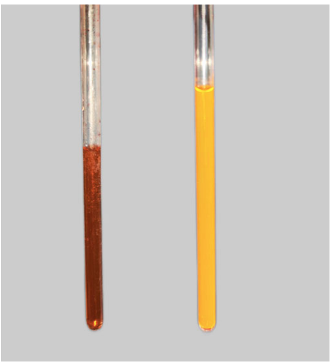

Figure S15. Color change before and after the addition of TFA-d; the left side shows the solution without TFA-d, and the right side shows the solution with TFA-d.

3. UV–vis Absorption Spectra

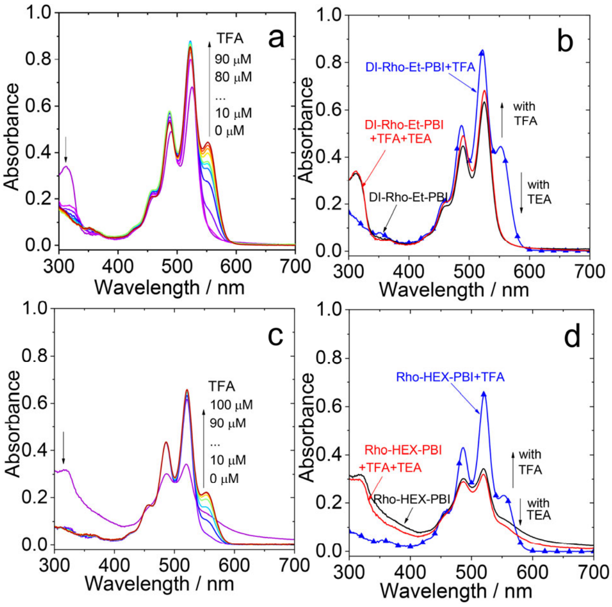

Figure S16. UV-vis absorption spectra of (a) **DI-Rho-Et-PBI** and (c) **Rho-HEX-PBI** with increasing amount of trifluoroacetic acid (TFA) added and (b) **DI-Rho-Et-PBI** (d) **Rho-HEX-PBI** with the addition of TFA or trimethylamine (TEA, neat, 300 μL) in MeOH. $c = 1.0 \times 10^{-5}$ M. 25 °C.

4. Fluorescence Spectra

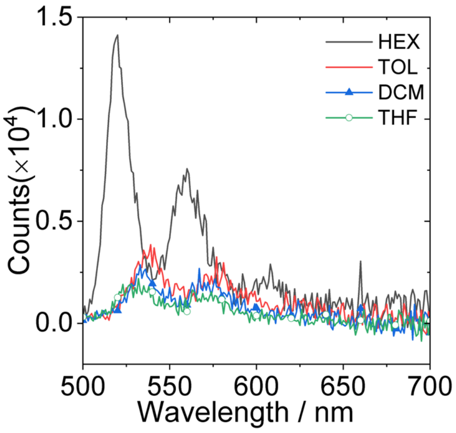

Figure S17. Comparison of fluorescence emission spectra of **Rho-HEX-PBI** in different solvents. λ_{ex} = 520 nm. $C = 1.0 \times 10^{-5}$ M. 20 °C.

5. Electrochemical study

$$E_{CS} = e[E_{OX} - E_{RED}] + \Delta G_S$$

$$\Delta G_S = -\frac{e^2}{4\pi\varepsilon_S\varepsilon_0 R_{CC}} - \frac{e^2}{8\pi\varepsilon_0}\left(\frac{1}{R_D} + \frac{1}{R_A}\right)\left(\frac{1}{\varepsilon_{REF}} - \frac{1}{\varepsilon_S}\right)$$

$$\Delta G^0{}_{CS} = e[E_{OX} - E_{RED}] - E_{00} + \Delta G_S$$

where ΔG_S is the static Coulombic energy. Among the Weller equation, e stands for electronic charge. E_{OX} stands for half-wave potential for one-electron oxidation of the electron-donor unit and E_{RED} is half-wave potential for one-electron reduction of the electron-acceptor unit. ε_S = static dielectric constant of the solvent, R_{CC} = center-to-center separation distance between the electron donor and acceptor, determined by results from theoretical calculations for structure optimization. R_D and R_A are the radius of the electron donor and acceptor, respectively. ε_{REF} is the static dielectric constant of the solvent used for the electrochemical studies, ε_0 is permittivity of vacuum. The solvents used in the calculations for free energy changes of the electron transfer process are toluene (ε_S = 2.38), dichloromethane (ε_S = 8.93) and acetonitrile (ε_S = 37.5)

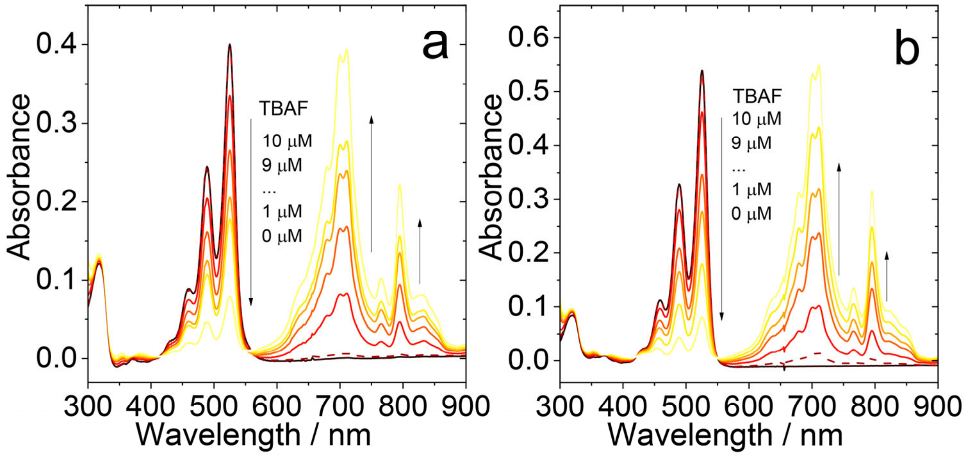

Figure S18. (a) **DI-Rho-HEX-PBI** and (b) **Rho-HEX-PBI** chemically reduced with tetrabutylammonium fluoride (TBAF) used to generate PBI$^{-\bullet}$ in deaerated DMF, 25 °C.

6. Nanosecond Transient Absorption Spectra

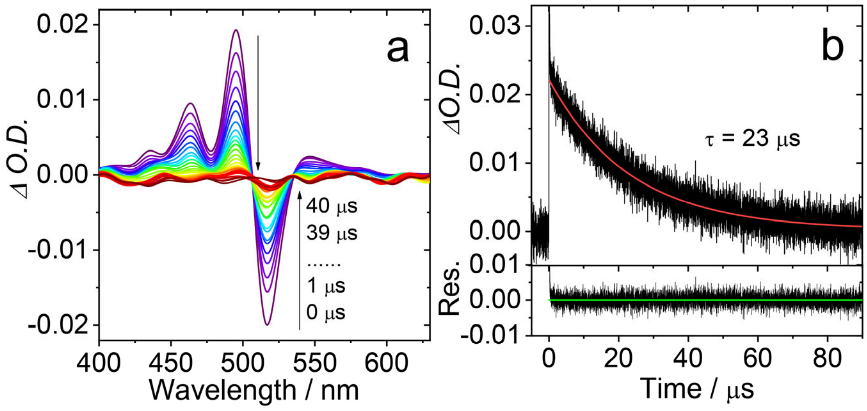

Figure S19. Nanosecond transient absorption spectra of **Rho-HEX-PBI** in silicone oil, c = 1.0 × 10^{-5} M. The decay trace of **Rho-HEX-PBI** at 505 nm. λ_{ex} = 520 nm. All solvents used in measurements are deaerated. 22 °C.

7. Femtosecond Transient Absorption Spectra

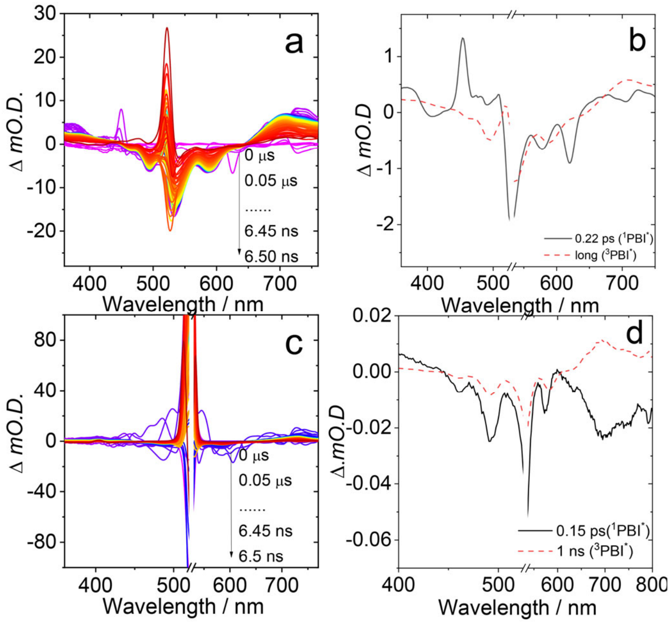

Figure S20. Femtosecond transient absorption spectra of **PBI-Br** in (a) TOL (c) ACN and the absorption spectra of (b) (d) in different solvents, λ_{ex} = 520 nm. c = 1.0 × 10^{-5} M, 25 °C.

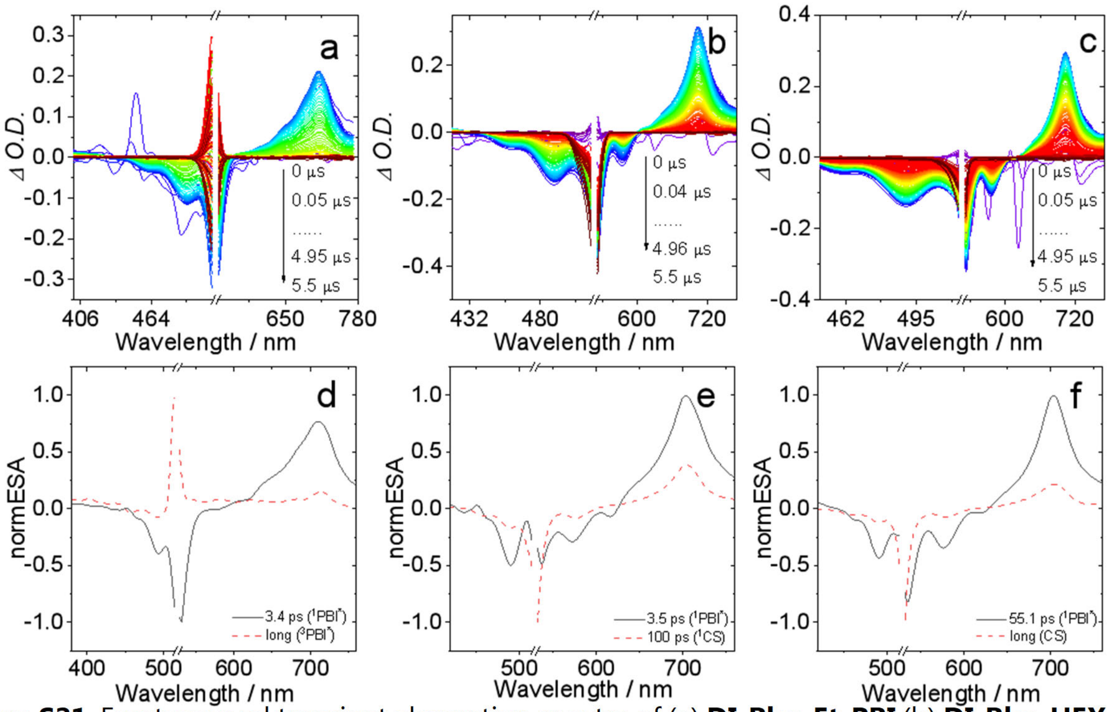

Figure S21. Femtosecond transient absorption spectra of (a) **DI-Rho-Et-PBI** (b) **DI-Rho-HEX-PBI** and (c) **Rho-HEX-PBI** in HEX, and the absorption spectra of (d) **DI-Rho-Et-PBI** (e) **DI-Rho-HEX-PBI** and (f) **Rho-HEX-PBI** in HEX, λ_{ex} = 520 nm. c = 1.0 × 10^{-5} M, 25 °C.

8. Photophysical Data of the Compounds.

Table S1. Photophysical Data of the Compounds.

Compounds	Solvent[a]	λ_{abs}/nm[b]	ε[c]	λ_{em}/nm[d]	Φ_F%[e]	Φ_Δ%[f]
**PBI-Br**	*n*-HEX	514	7.4	525	98.24	7.5
	DCM	524	6.8	538	95.31	6.7
	ACN	495	2.4	533	98.52	17.5
**DI-Rho-Et-PBI**	*n*-HEX	499	1.8	517	0.51	1.3
	DCM	526	6.4	536	0.21	0
	ACN	520	5.9	534	0.26	0
**DI-Rho-HEX-PBI**	*n*-HEX	496	1.7	519	0.0024	1.1
	DCM	523	5.9	532	2.51	0
	ACN	518	4.9	528	1.56	0
**Rho-HEX-PBI**	*n*-HEX	513	2.9	520	2.02	2.7
	DCM	523	7.5	534	13.63	0
	ACN	519	5.3	531	6.04	0

[a] In different solvents (c = 1.0 × 10^{-5} M). [b] Significant UV–vis absorption wavelength, c = 1.0 × 10^{-5} M, 20 °C. [c] Molar absorption coefficient, ε: 10^4 M^{-1} cm^{-1}. [d] Fluorescence emission wavelength. [e] Absolute fluorescence quantum yields. [f] Quantum yield of singlet oxygen, in%. [g] 2,6-diiodoBodipy (**2I-BDP**) was used as standard compound (Φ_Δ = 87% in DCM), λ_{ex} = 520 nm.

9. DFT/TDDFT Calculations.

Table S2. SOCMEs (cm^{-1} units) in the Gas Phase for the State Pairs Evaluated at the Optimized Geometry of the Ground State.[a]

Compounds	ISC	SOCMEs	ΔE [b]
**DI-Rho-Et-PBI**	$S_1 \rightarrow T_1$	0.03	0.14
	$S_1 \rightarrow T_2$	0	0.00
	$S_1 \rightarrow T_3$	0	−0.10
	$S_1 \rightarrow T_4$	1.11	−0.13
**DI-Rho-HEX-PBI**	$S_1 \rightarrow T_1$	0	0.13
	$S_1 \rightarrow T_2$	0	0.00
	$S_1 \rightarrow T_3$	0	−0.02
	$S_1 \rightarrow T_4$	1.26	−0.13
**Rho-HEX-PBI**	$S_1 \rightarrow T_1$	0	0.24
	$S_1 \rightarrow T_2$	0	0.00
	$S_1 \rightarrow T_3$	1.21	−0.11
	$S_1 \rightarrow T_4$	0.09	−0.66

[a]The calculation of the spin orbital coupling matrix elements (SOCMEs) were performed at CAM-B3LYP/def2-SVP level using the ORCA program. [b] $\Delta E = E(S_1) - E(T_m)$.

Table S3. Zero Field Splitting Parameters (D and E), Spin−Spin and Spin−Orbit (cm^{-1}) in ACN for the State Pairs Evaluated at the Optimized Ground State.[a]

Compounds	ZFS		SPIN−SPIN		SPIN−ORBIT	
	D / cm^{-1}	E / cm^{-1}	D / cm^{-1}	E / cm^{-1}	D / cm^{-1}	E / cm^{-1}
**DI-Rho-Et-PBI**	0.054	0.008	0.001	−0.001	0.053	0.009
**DI-Rho-HEX-PBI**	0.060	0.009	0.000	0.000	0.060	0.009
**Rho-HEX-PBI**	0.053	0.009	0.000	0.000	0.053	−0.010

[a] The calculation of the zero field splitting parameters (ZFS), spin−spin and spin−orbit were performed at CAM-B3LYP/EPR-II level (acetonitrile) using the ORCA 6.0 programs.
